# Effects of Active Paper Sheets on the Quality of Cherry Tomatoes and Kale During Storage

**DOI:** 10.3390/foods14244225

**Published:** 2025-12-09

**Authors:** Alejandra Navarro-Martínez, Yineth Piñeros-Castro, Alberto Garre, Antonio López-Gómez, Ginés Benito Martínez-Hernández

**Affiliations:** 1 Food Safety and Refrigeration Engineering Group, Department of Agricultural Engineering, Universidad Politécnica de Cartagena, Paseo Alfonso XIII, 48, 30203 Cartagena, Spain; alejandra.navarro@upct.es (A.N.-M.); antonio.lopez@upct.es (A.L.-G.); 2 Academic Area of Sustainable Process and Products, Natural Sciences and Engineering Faculty, Universidad Jorge Tadeo Lozano, Cra. 4 22-61, Bogotá, Colombia; yineth.pineros@utadeo.edu.co; 3 Department of Agricultural Engineering, Universidad Politécnica de Cartagena, Paseo Alfonso XIII, 48, 30203 Cartagena, Spain; alberto.garre@upct.es; 4 Institute of Plant Biotechnology, Campus Muralla del Mar (Universidad Politécnica de Cartagena), 30202 Cartagena, Spain

**Keywords:** cyclodextrin inclusion complex, plant essential oils, active packaging, Baranyi-Ratkowsky, quality, colour, phenolic compounds, vitamin C, antioxidant

## Abstract

The effect of active packaging on maintaining the quality of cherry tomatoes and kale during storage was investigated. The active packaging consisted of kraft paper sheets coated with thymol/eugenol (50:50) encapsulated in β-cyclodextrin. Cherry tomatoes were stored at 10, 15, and 22 °C for 15, 14, and 8 days, respectively, while kale was stored at 2, 8, 15, and 22 °C for 21, 16, 9, and 7 days. Physicochemical (pH, total soluble solids, titratable acidity, colour, and firmness), microbiological (mesophilic, psychrophilic, enterobacteria, moulds, and yeasts) and pigment/bioactive/nutritional (chlorophylls, carotenoids, total phenolic content, vitamin C, and total antioxidant capacity) characteristics were analysed. Active packaging significantly reduced microbial growth, particularly enterobacteria and moulds, in cherry tomatoes and psychrophiles and moulds in kale, without negatively affecting the physicochemical quality. The microbial kinetics were successfully described using the Baranyi–Ratkowsky predictive model, which quantified the effects of temperature and active packaging on microbial growth parameters. This modelling approach revealed that active packaging increased the minimum growth temperature and reduced the specific growth rate of key microbial groups, confirming its inhibitory action under different storage conditions. The use of active packaging slowed colour degradation in kale by reducing chlorophyll loss up to 50% at 22 °C and maintained tomato firmness and colour during storage. Furthermore, a strong correlation (R^2^ = 0.87) between colour index and carotenoid content was found, enabling the non-destructive prediction of ripening in tomatoes. Overall, active packaging enhanced microbial stability, delayed visual deterioration, and sustainably extended the shelf life and post-harvest quality of perishable products, offering a promising alternative to conventional preservation methods.

## 1. Introduction

Tomatoes of the cherry type (*Solanum lycopersicum* var. *cerasiforme*) are a fruit vegetable that is within the group of vegetables promoted by FAO [1]. They are highly rich in nutritional and bioactive compounds with high health-promoting properties [2]. In particular, tomatoes have high levels of carotenes, mainly lycopene and β-carotene, whose properties include the prevention of prostate cancer and cardiovascular risks, etc. [3]. Tomatoes are also a rich source of vitamin C, which has a high antioxidant capacity and prevents coronary heart disease and cancer of the liver, stomach, or lung [4]. Total vitamin C is composed of ascorbic acid and dehydroascorbic acid, since it may also be absorbed and converted back into ascorbic acid in the human body. Other nutritional and bioactive compounds of tomatoes include vitamin E, potassium, folic acid, and phenolic compounds, etc. [2]. The tomato quality is affected during its post-harvest life, in particular, their soluble solid content, pH, titratable acidity, firmness, colour and sensory qualities are altered [5]. In particular, the cell wall and the pectin and cellulose content of the intermediate and primary laminae are affected at higher storage temperatures, resulting in reduced tomato firmness [6]. In addition, the enzymatic activity of pectin-methyl-esterase and polygalacturonase, among others, causes the loss of water and consequent deterioration of the structures, which, apart from the quality reduction, generates economic losses for the food industry [7]. Vitamin C in tomatoes is also altered during the post-harvest life, with losses of ≥30%. *Botrytis cinerea* is mainly responsible for the occurrence of tomato quality reduction and the consequent economic loss. For this, different post-harvest preservation techniques have been developed, such as low temperature storage, high relative humidity and modified atmospheres, and edible coatings, etc. [8].

The consumption of kale (*Brassica oleracea* var. *sabellica*), a leafy winter vegetable of the *Brassicaceae* family, is currently on the rise. There are several types of commercial kale with differences in leaf shape or colour, such as purple or scarlet kale [9]. Kale is rich in glucosinolates, vitamin C (in addition to A, B1, B2, B6, E, folic acid, and niacin), minerals (K, Ca, Mg, Fe, and Cu), phenolic compounds (quercetin and kaempferol), flavonoids, proteins, and fibre [10,11,12]. Such nutritional and bioactive compounds of kale are reduced during its post-harvest life [9]. Among the main post-harvest quality degradation symptoms of kale are yellowing, which is related to the degradation of chlorophylls. In addition, the nutritional and bioactive quality may be highly altered during post-harvest life [9]. However, low storage temperatures between 0 and 5 °C, together with high relative humidity, have been conventionally recommended [13]. Nevertheless, there is a need to find new economical and viable post-harvest technologies to increase the product’s shelf life and preserve the quality of this vegetable.

Antimicrobial active packaging with plant essential oils (EOs) is an environmentally friendly alternative for the post-harvest preservation of plant products and a way to increase shelf life and preserve quality by avoiding the addition of chemical and health-damaging products to food. EOs have high antimicrobial, antioxidant, and enzyme-inhibitory properties, among others, provided by their constituent terpenes, terpenoids, aromatic, and low-molecular-weight aliphatic chemicals [14]. However, some factors indeed affect the efficacy of these EOs due to evaporation processes, degradation reactions (by oxidation and heat, etc.). Therefore, EO encapsulation within cyclodextrins forming inclusion complexes will allow a controlled EO release over time and protect them from degradation. The release of encapsulated EOs is activated by high relative humidities, such as those of the cold chambers of the horticultural facilities. Furthermore, it is crucial to use sustainable packaging materials such as cardboard or kraft paper. Thus, active packaging made of paper or cardboard containing encapsulated EOs represents a high-potential post-harvest approach to reduce food waste, while also aligning with the principles of the circular economy. In this context, our research group has studied the effect of active paper/cardboard packaging on extending the shelf life of various products such as lemons, flat peaches, lettuce, grapes, nectarines, mandarins, and bell peppers. However, until now, a sustainable active paper packaging containing encapsulated thymol and eugenol has not been studied for kale and cherry tomatoes.

The study aimed to evaluate the microbiological and physicochemical quality and the content of bioactive compounds (vitamin C, carotenes, total phenolic compounds, and antioxidative activity) of cherry tomatoes preserved with active packaging compared to the control packaging at three storage temperatures (10, 15, and 22 °C for 15, 14, and 8 days, respectively) and kale stored at four preservation temperatures (2, 8, 15, and 22 °C for 21, 16, 9, and 7 days, respectively).

## 2. Materials and Methods

### 2.1. Materials

Thymol (99.5% purity) and eugenol (99.5% purity) were encapsulated in β-cyclodextrin (hereafter referred to as βCD, and provided by Roquette -Klep-tose^®^10; Lestrem, France) at an industrial scale by the company Bioencapsulation and iPackaging S.L. (Bio-iPack, Fuente-Álamo, Spain). Water-based emulsion, including the inclusion complex (and without wax or acrylic polymer; with pH 4–6; viscosity max. 80 mPa s at 20 °C; with 24% total solids concentration), was manufactured by Bio-iPack, and applied to recycled kraft paper sheets (50 g cm^−2^) by the company Papeles El Carmen SAU (Navarra, Spain). All chemical materials (unless otherwise specified) were acquired from Merck (Berlin, Germany), and the microbiological supplies from Scharlau (Barcelona, Spain).

The cherry-type tomatoes (*Solanum lycopersicum* var. *cerasiforme* ‘Uvalina’) and kale (*Brassica oleracea* var. *sabellica*) were produced and provided by the Biosystems centre of the Universidad Jorge Tadeo Lozano (Bogotá, Colombia) in November 2023 and January 2024, respectively. Both vegetables were grown in the greenhouses of the Biosystems centre.

### 2.2. Encapsulation of Essential Oils and Active Packaging Preparation

Thymol and eugenol were selected based on their high capacity to inhibit ethylene production and extend the shelf life of these products, based on preliminary experiments with approximately 50 different EOs [15].

The EO−βCD inclusion complex was prepared using the kneading method [16]. Briefly, each 1 g of EO mix (thymol/eugenol 50:50 weight (*w*/*w*)) was mixed with 7.6 g of βCD (following a 1:1 EO/βCD molar ratio) in a mixer with 3 mL of ethanol, kneaded for 45 min, and finally maintained in vacuum at room temperature for at least 72 h (it evaporates the thymol–eugenol not encapsulated within the βCD). The EO−βCD inclusion complex was incorporated into the water-based emulsion and then applied by flexography (at an industrial level in the Papeles El Carmen company) on the kraft paper. The water-based emulsion was prepared to a final solids concentration of 24% to control the viscosity increase due to the addition of the EO−βCD inclusion complex, as a water-based emulsion with a solid content of ≥24% may be difficult to apply on the paper surface by flexography. The active paper sheet was prepared and protected within a sealed plastic bag till posterior use.

The final EO−βCD inclusion complex load on the paper was 1000 mg m^−2^ (as previously optimised [15]), which would be equivalent (based on the previous EOs:βCD molar ratio of 1:7.6) to entrapping 116.3 mg of thymol/eugenol mix per m^2^ of paper. The selected load range of the EO−βCD inclusion complex was selected based on our previous publication [15]. Active packaging material was prepared one day before the experiments.

### 2.3. Packaging Treatments and Storage Conditions

Tomatoes and kale were previously sanitised using a NaOCl wash (100 mg L^−1^; 1 min; pH 6.5), followed by a 1 min rinse with tap water. Subsequently, kale (≈70 g) and tomatoes (≈150 g) were placed inside a zip-lock plastic bag (150 × 150 mm for tomatoes and 270 × 300 mm for kale) (Mercadona; Valencia, Spain). For active packaging, one active paper sheet (150 × 150 mm) was introduced in each bag containing plant products, while a paper sheet without the inclusion of the EO−βCD complex was considered as the control packaging treatment. Packaged tomatoes were stored at 10, 15, and 22 °C, while kale was stored at 2, 8, 15, and 22 °C. Tomatoes are classified as chilling-sensitive, occurring chilling injury below 10–12 °C (depending on the storage time) [17,18]. Therefore, storage at 10–12 °C was selected as a typical commercial range that slows respiration and decay while posing minimal risk of chilling injury within normal marketing periods. The sampling of tomato samples was performed after 15, 14, and 8 days at 10, 15, and 22 °C, respectively; while the sampling of kale samples was performed after 21, 16, 9, and 7 days at 2, 8, 15, and 22 °C, respectively. Five tomatoes per packaging treatment, storage temperature, and sampling time were used as replicates.

Analyses and determinations (described in the following sections) were made at each sampling time, while samples for pigments/bioactive/nutritional compounds (carotenoids, chlorophylls, vitamin C, phenolic compounds, and antioxidant capacity) were kept at −80 °C until analyses.

### 2.4. Microbiological Analyses

Microbiological analyses were conducted as previously described [19], with slight modifications. For this, plant product (4 tomatoes or 2 kale leaves) was mixed in sterile plastic bags (Seward; Worthing, United Kingdom) with buffered peptone water (1:10 *w*:*v*) and shaken in an orbital shaker (Stuart model SSL1; Staffordshire, UK) at 120 rpm for 1 h (4 °C) to obtain the microbial suspension. The needed 1:10 dilutions of the microbial suspension were then made with buffered peptone water. Then, deep agar inoculation (1 mL of the microbial suspension) was performed in Petri dishes using the corresponding agar media for mesophiles (plate count agar), psychrophiles (plate count agar), and enterobacteria (Violet Red Bile Glucose Agar) and incubated at 31 °C/48 h, 4 °C/7 days, and 37 °C/24 h, respectively. For yeast and moulds, surface plating (0.1 mL of the microbial suspension) on rose bengal agar was performed and incubated at 25 °C for 5 and 7 days, respectively. Results were expressed as the log of colony-forming units (CFUs) per gram of sample (log CFU g^−1^). Each of the samples (packaging treatment × storage temperature × storage time × 3 replicates) was analysed in duplicate.

### 2.5. Physicochemical and Firmness Analyses

Total soluble solids, pH, and total titratable acidity were determined on the prepared product juice. For this, the juice of five tomatoes or five kale leaves was obtained using a domestic food grinder (DuraPro; BLACK + DECKER; Reynosa, Mexico) and, subsequently, was manually filtered with a 4-layer cheesecloth. The juice was previously diluted (1 mL of juice and 5 mL of distilled water). The total soluble solid content of the juice was determined using a digital refractometer (model Pocket PAL-1, Atago; Tokyo, Japan) at room temperature, previously calibrated with distilled water, and the results were expressed in °Brix. The pH was also determined on the juice with a pH meter (model Basic 20, Crison; Barcelona, Spain) together with the previous calibration of the equipment (pH standards of 4 and 7). The titratable acidity (TA) was also measured in the juice by titration with 0.1 N NaOH to a pH of 8.1 (monitored with the pH meter) and expressed as % citric acid. Firmness measurements of tomatoes were performed with a penetrometer (Chatillon DFM-100, AMETEK Test and Calibration Instruments; Largo FL, USA) using the force required (N) to penetrate the surface of the tomato with a cylindrical test tube of 0.4 mm diameter.

### 2.6. Colour

Colour was analysed with a colourimeter (Chrome Meter CR-400; Konica Minolta; Tokyo, Japan) with D65 illuminant, 2° observer and 8 mm observation aperture. The colourimeter was previously calibrated with the standard reflective plate. Three measurements were taken from each sample replicate and automatically averaged by the colourimeter. Then, the following colour indexes were calculated with the L*, a*, and b* measurements from the colourimeter:

(1) $$Chroma=\sqrt{a^{*2}+b^{*2}}$$

(2) $$Hue=arctan\frac{b^{*}}{a^{*}}$$

(3) $$Colur\;Index=\frac{\;2000\;\times \;a^{*}}{L^{*}\times \;Chroma}$$

(4) $$Yellowness\;Index=\frac{\;14.286\;\times \;b^{*}}{L^{*}}$$

(5) $$Total\;colour\;Difference=\sqrt{{\left(a^{*}-a_{0}^{*}\right)}^{2}+{\left(b^{*}-b_{0}^{*}\right)}^{2}+{\left(L^{*}-L_{0}^{*}\right)}^{2}}$$

where a*, b*, and L* are the colour parameters measured at each sampling time, while *a*_0_*, *b*_0_*, and *L*_0_* are the corresponding values at day 0.

### 2.7. Pigment Analyses

For the analysis of chlorophylls and carotenoids, 2 g of the stored frozen (previously ground using liquid N_2_) samples were mixed with 3.6 mL of hexane and 4.2 mL of an acetone/methanol (2:1, *v*:*v*) mix. It was then shaken in the orbital shaker for 4 h at 200 rpm at 4 °C. Then, 10 mL of 1 M NaCl was added, vortexed and centrifuged at 4000× *g* for 10 min. Finally, the upper layer (hexane) was collected and used as the carotenoids/chlorophyll extract. Finally, absorbances of extracts were measured at 470, 662, and 644 nm in a spectrophotometer. Chlorophylls (a and b) and total carotenoids (carotenes and xanthophylls) were calculated according to Equations (6)–(8) [20], and the concentration results obtained from those equations (in µg mL^−1^) were then converted to mg kg^−1^ on a fresh-weight (FW) basis.

(6) $$C_{a}=\left(10.05\times A_{662}\right)-\left(0.766\times A_{644}\right)$$

(7) $$C_{b}=\left(16.37\times A_{644}\right)-\left(3.14\times A_{662}\right)$$

(8) $$Carotenoids=\frac{\left(1000\times \;A_{470}\right)-\left(1.28\times C_{a}\right)-\left(56.7\times C_{b}\right)}{205}$$

### 2.8. Total Vitamin C Content

The total vitamin (ascorbic and dehydroascorbic acids) was measured following the procedures previously described by [21,22], with minor modifications according to [23]. In brief, 5 g of ground frozen sample was mixed with 10 mL of a cold extraction buffer (0.1 M citric acid, 1.3 mM ethylenediaminetetraacetic acid, 4 mM sodium fluoride, and 5% methanol) and homogenised at 10,000 rpm for 10 s using an Ultraturrax (IKA T-18 digital, ULTRA-TURRAX^®^; Königswinter, Germany). The pH of the extract was adjusted to 2.35–2.40 and filtered through a 4-layer cheesecloth. The filtered extract was then purified with solid-phase extraction cartridges (Sep-Pak C18, Waters; Dublin, Ireland) and filtered through a 0.45 μm polyethersulfone membrane before HPLC analysis.

The vitamin C extract was analysed using an HPLC system (Series 1100, Agilent Technologies; Waldbronn, Germany). Prior to analyses, extracts were derivatisated by mixing 750 μL of the extract with 250 μL of 7.7 M 1,2-phenylenediamine in an amber HPLC vial and the reaction was allowed to proceed for 37 min at room temperature. Afterwards, 20 μL of the derivatised sample was injected into a Gemini NX C18 column (250 mm × 4.6 mm, 5 μm, Phenomenex; Torrance, CA, USA), using mobile phase (5 mM hexadecyltrimethylammonium bromide, 50 mM KH_2_PO_4_, and 5% methanol; pH 4.59) at 1.8 mL min^−1^ and 25 °C. Chromatograms were recorded at 261 nm (ascorbic acid) and 348 nm (dehydroascorbic acid) for 14 min. Quantification was performed using commercial HPLC-grade standards of ascorbic and dehydroascorbic acids (Sigma; St. Louis MO, USA). The total vitamin C content (sum of ascorbic acid and dehydroascorbic acid contents) was expressed as mg kg^−1^ on a FW basis.

### 2.9. Total Phenolic Content and Total Antioxidant Capacity

A common extract for phenolic compounds and total antioxidant capacity was made as previously described. Briefly, 5 g of ground frozen sample was mixed with 20 mL of extraction buffer (methanol/water 80:20 (*v*:*v*) and 2 mM sodium fluoride) and homogenised for 10 s with the Ultraturrax at 10,000 rpm. Extracts were then shaken for 1 h at 120 rpm and 4 °C in the orbital shaker. Finally, they were centrifuged at 14,000× *g* for 10 min at 4 °C, and the supernatants were used as the common extracts for phenolic compounds and total antioxidant capacity analyses. Analyses of total phenolic content and total antioxidant capacity were performed according to the methods described by [24] and [25], respectively.

For the phenolic analysis, 19 μL of diluted extract (30% and 5% for tomato and kale, respectively) was placed in a well of a 96-well plate (Greiner-Bio-one; Frickenhaus, Germany) and 29 μL of 1 N Folin–Ciocalteu was added and allowed to react for 3 min in darkness. Then, 192 μL of saline buffer (0.1 M NaOH and 0.19 M Na_2_CO_3_ in ultrafiltered water) was added and allowed to react in the dark for 2 h. Finally, the absorbance was measured at 750 nm with a microplate reader (Infinite M200, Tecan; Männedorf, Switzerland). Quantification was performed using a commercial gallic acid standard (Sigma, St. Louis, MO, USA) and expressed as equivalents of gallic acid in mg kg^−1^ on a FW basis.

For the antioxidant analysis, 21 μL of diluted extract (30% and 5% for tomato and kale, respectively) was placed in a well of a 96-well plate (Greiner-Bio-one; Frickenhaus, Germany) and 194 μL of pre-adjusted (to 1.10 ± 0.02 absorbance at 517 nm) DPPH (2,2-diphenyl-1-picrylhydrazyl) methanolic solution was added and allowed to react in the dark for 20 min. Finally, the absorbance was measured at 517 nm with the microplate reader. Quantification was performed using a commercial Trolox (6-hydroxy-2,5,7,8-tetramethylchroman-2-carboxylic acid) standard (Sigma, St. Louis, MO, USA) and expressed as equivalents of Trolox in mg kg^−1^ on FW basis.

### 2.10. Mathematical Modelling of Microbial Response

The microbial response was described using the Baranyi–Ratkowsky model, commonly used in predictive microbiology [26]. This model describes the changes in microbial concentration (*N*) with respect to storage time (*t*) using a sigmoidal model defined by Equation (9) [27]. The relationship is scaled by the lag phase duration (λ), the maximum specific growth rate (μ), the initial microbial concentration ($N_{0}$), and the maximum concentration in the stationary growth phase ($N_{max}$).

(9) $$\ln N=\ln N_{0}+\mu_{max}A\left(t\right)-\ln\left(1+\frac{e^{\mu_{max}A(t)}-1}{e^{\ln N_{max}-\ln N_{0}}}\right)$$

$$A\left(t\right)=t-\lambda +\frac{1}{\mu_{max}}\ln\left(1-e^{-\mu_{max}t}+e^{-\mu_{max}\left(t-\lambda \right)}\right)$$

The sub-optimal Ratkowsky model [28] assumes a linear relationship between $\sqrt{\mu }$ and the storage temperature (T). As defined in Equation (10), this model introduces a theoretical minimum temperature for growth ($T_{min}$), as well as a slope term b.

(10) $$\sqrt{\mu_{max}}=b\left(T-T_{min}\right);T>T_{min}$$

$$\sqrt{\mu_{max}}=0;\mathrm{otherwise}$$

A recent study [29] demonstrated that only an inverse square-root secondary model for λ is compatible with the assumptions of the Baranyi–Ratkowsky model (Equation (11)). Furthermore, this model would be coupled with the secondary model, introducing a single additional parameter, $C_{0}$, related to the lag phase duration and presumably representing the physiological state of the cells.

(11) $$\frac{1}{\sqrt{\lambda }}=\frac{1}{\sqrt{B}}(T-T_{min})$$

$$B=\frac{\ln\left(1+\frac{1}{C_{0}}\right)}{b^{2}}$$

The Baranyi–Ratkowsky model was fitted to each condition independently (combination of product, microorganism, and type of packaging). The model was fitted using a one-step algorithm (i.e., fitting both primary and secondary models in a single step), as this approach has proven to be more robust from a statistical viewpoint [30]. Accordingly, the microbial response for each condition (and its relationship with temperature) would be described by five model parameters ($N_{0},N_{max},b,T_{min},\;\mathrm{and}\;C_{0}$). The parameters were estimated using the functions included in the *biogrowth* package for R [31], applying a log-transformation to parameters $N_{0},N_{max},\;\mathrm{and}\;C_{0}$ for better parameter identifiability.

As some conditions did not show a clear lag phase, the Baranyi–Ratkowsky model was also fitted, fixing $C_{0}=10^{8}$, representing a situation without lag. The reduced model was compared against the complete one based on the Akaike Information Criterion (*AIC*), commonly used for model selection, reporting only the best model.

### 2.11. Data Analysis for Quality Parameters

The relationships between the various quality parameters of tomato or kale were analysed using classical statistical methods. First, the datasets were explored based on the Spearman correlation index to identify parameters with high correlations. This index was selected over Pearson’s due to Spearman’s being more robust against non-linear relationships.

The descriptive analysis showed that the carotene content of tomatoes had a high association with the colour index. Considering that the latter is a cost-effective, non-destructive method, a linear regression model was used to predict the carotene content from the colour index. All calculations were performed in R version 4.2.3 [32]. The code is available in open access from the GitHub page of one of the co-authors (https://github.com/albgarre/active-sheets-tomato-kale (accessed on 1 September 2025).

## 3. Results and Discussion

### 3.1. Microbiological Analysis

The initial microbial loads in cherry tomato for mesophilic, psychrophilic, enterobacteria, and yeasts were 2.85, 3.03, 2.95, and 3.02 log CFU g^−1^, respectively, while moulds were below the detection limit (<2 log CFU g^−1^) (Supplementary Material Figure S1). The microbial growth was higher (*p* < 0.05) at higher storage temperatures, with increments of 1.4–2.7, 2.0–3.1, and 2.7–4.8 log units (ranges including all microbial groups) at the end of storage periods at 10, 15, and 22 °C (for control samples), respectively. Active packaging reduced microbial growth during storage.

The Baranyi–Ratkowsky model was successful at describing the microbial response (Supplementary Material Figure S1), based on the model parameters reported in Table 1 (A). The only exception was the psychrophiles under active packaging. This is due to no growth being observed at 10 or 15 °C, so the data does not contain enough information to estimate the parameters of the secondary model reliably, resulting in high parameter uncertainty. Nonetheless, the model still provides valuable information for summarising the microbial response, showing that the active packaging inhibits growth by raising the minimum temperature for growth (parameter $T_{min}$).

**Figure 1:**
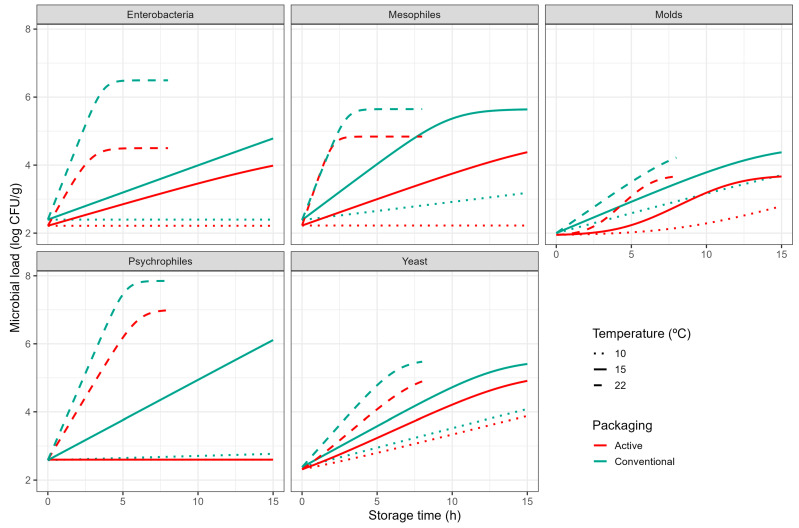
Growth of different microbial groups on fresh cherry tomato packaged under control packaging (blue) or active packaging (red) based on the Baranyi–Ratkowsky model during storage at 10 (· ·), 15 (-), and 22 °C (- -).

Microbial growth was described using a global fitting approach to improve the robustness of parameter estimates. On the other hand, the interpretation of global models is more complicated [33], so Figure 1 includes the fitted growth curves to support interpretation. It shows that microbial growth under active packaging was inhibited with respect to control conditions. Depending on the microbial species, the inhibition was due to (1) an increase in the lag phase duration, (2) a reduction in the specific growth rate, (3) a reduction in the maximum concentration in the stationary phase, or a combination of the two.

Active packaging inhibited the growth of moulds on tomatoes through the introduction of a lag phase that did not appear under control conditions. This is underlined by the estimate of parameter $\log C_{0}$ ($\log C_{0}=1.34\pm 0.4$), whereas control conditions had no lag. Considering that the lag phase represents a need for microbial cells to adapt to the environment [34]. Active packaging would make it less favourable for mould growth, ultimately extending shelf life.

The growth of yeast was also inhibited by the active packaging through the introduction of a lag phase. However, the estimates of $\log C_{0}$ for yeast ($\log C_{0}=1.34\pm 0.4$) were lower than for moulds. Instead, the inhibition of yeast growth is mainly due to the reduction in the minimum temperature for growth ($T_{min}$ of −4.4 °C under active packaging; −1.09 °C under conventional packaging). As there are no significant differences in the slope parameter of the secondary model (b) between both conditions, this results in an overall reduction in the specific growth rate (μ) through the complete temperature range.

The growth inhibition of psychrophiles is especially noticeable, as they were only able to grow at 22 °C under active packaging, while the population could develop at 15 °C under control conditions. This is reflected in a very high estimate for parameter $T_{min}$ under active packaging (20 °C). However, the parameter estimates for this particular model should be taken with care, as they are based on a single growth curve (Figure 1). This is reflected in the very high parameter uncertainty for these parameters. Nonetheless, this high uncertainty supports our interpretation that the growth of psychrophiles was largely inhibited by the active packaging, resulting in no noticeable growth under most conditions.

The active packaging also inhibited the growth of enterobacteria at every temperature tested. In this case, the minimum temperature for growth was not affected (no significant differences in the $T_{min}$ estimates). Instead, the inhibition is reflected in the estimates for parameter b (0.11 °C^−1^ in active packaging, 0.14 °C^−1^ in conventional). As this parameter reflects the slope of the secondary model for μ, a reduction in this parameter estimate implies a lower growth rate during the exponential phase, with the differences with respect to control conditions becoming larger as the temperature increases. This is clearly illustrated in Figure 1, with growth inhibition of enterobacteria being more noticeable at 22 °C than at 15 or 10 °C.

Growth inhibition for mesophiles is harder to interpret from parameter estimates, as there are differences in both $T_{min}$ and b. From Figure 1, it is evident that active packaging inhibited mesophile growth at 10 and 15 °C. However, the specific growth rate observed at 22 °C was practically the same in both packaging conditions. Regardless, the maximum concentration during the stationary phase was reduced by almost one log unit (log $N_{max}$ of 5.65 vs. 4.84 log CFU/g), demonstrating the effectiveness of the active packaging at inhibiting microbial growth on tomatoes in every condition tested.

For kale, initial microbial loads for mesophilic, psychrophilic, enterobacteria, moulds, and yeasts were 3.11, 3.41, 2.01, 2.15, and 2.40 log CFU g^−1^ (Supplementary Material Figure S2). The microbial growth was higher at higher storage temperatures, with increments of 2.9–5.3 at 2 °C to 4.2–6.8 at 22 °C log units (ranges including all microbial groups) at the end of the storage periods for control samples. Active packaging also reduced microbial growth in kale during storage. In particular, the highest reductions for mesophilic, psychrophilic, enterobacteria, moulds, and yeasts were observed at 8 °C, 22 °C, 2 °C, 2–15 °C, and 8 °C, respectively, with log unit decreases of 0.7, 1.4, 1.3, 0.8–1.3, and 0.8, respectively, compared to control samples at the end of the corresponding storage times.

**Figure 2:**
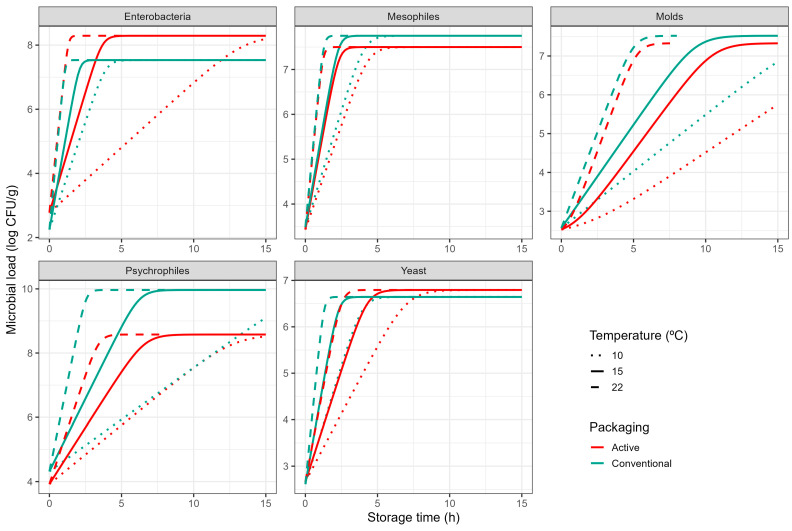
Growth of different microbial groups on fresh kale packaged under control packaging (blue) or active packaging (red) based on the Baranyi–Ratkowsky model during storage at 10 (· ·), 15 (-), and 22 °C (- -).

In a similar way to the data on tomato, the Baranyi–Ratkowsky model was successful at describing microbial growth for all conditions tested (Supplementary Material Figure S2) based on the parameters reported in Table 1 (B). As illustrated in Figure 2, active packaging inhibited microbial growth in most conditions, although the magnitude was different from that observed in tomato (Figure 1). As well as in tomato, the active packaging induced a lag phase in mould growth on kale, although the lag phase was generally smaller ($\log C_{0}=0.35$). Therefore, active packaging would introduce some hurdle for mould growth that must be overcome before exponential growth can start. The active packaging also increased the minimum temperature for growth of moulds ($T_{min}$ of −1.8 °C against −4.3 °C), resulting in an additional inhibition on the growth rate during the exponential phase.

Active packaging had a clear inhibitory effect on the growth of psychrophiles on kale. The maximum concentration during the stationary phase was reduced by 1.5 log cycles ($\log N_{max}$ estimates of 8.58 log CFU/g vs. 9.97 log CFU/g). The specific growth rate during the exponential phase was also reduced, although estimates of $T_{min}$ were lower in active packaging than in control conditions. This is due to this parameter being an extrapolation of the secondary model that should not be generally interpreted as a growth limit. Nonetheless, due to the estimate of b being much lower in active packaging (0.07 °C^−1^) than under conventional conditions (0.12 °C^−1^), the active packaging has a clear inhibitory effect, especially at higher temperatures (Figure 1).

The experiments reflect that active packaging lowers the growth of yeasts on kale under all conditions tested. This is due to a reduction in both the estimates of $T_{min}$ (−7.81 °C vs. −6.10 °C) and b (0.07 °C^−1^ vs. 0.10 °C^−1^). As a result, the specific growth rate of yeast during the exponential phase is clearly reduced through the entire biokinetic range (Figure 1).

On the other hand, active packaging did not influence the growth kinetics of mesophilic bacteria on kale (no significant differences between parameter estimates based on *t*-test at α=0.05). It also had a relatively minor impact on enterobacteria growth kinetics. Although there was a reduction in $T_{min}$ (−4.59 °C for active packaging vs. −3.47 °C under control conditions), active packaging conditions have a higher slope term (b estimates of 0.18 °C^−1^ vs. 0.13 °C^−1^). As a result, the inhibition during the exponential phase is evident at low temperatures (Figure 1), but the growth kinetics are mostly identical at the highest temperature tested (22 °C). Furthermore, the maximum concentration during the stationary phase for enterobacteria on kale was higher under active packaging (8.29 log CFU/g) than for conventional packaging (7.53 log CFU/g). This could be due to the inhibition of other bacterial groups freeing an ecological niche that is occupied by enterobacteria. Nonetheless, the relevance of this result for product shelf life is relatively minor, as the maximum microbial concentrations under both conditions are above the level that is generally considered to cause consumer rejection.

In conclusion, active packaging with EOs effectively reduced microbial growth in cherry tomato and kale during storage. Its antimicrobial efficacy depended on temperature, showing stronger effects against bacteria at higher temperatures and against moulds at lower temperatures. This temperature-dependent behaviour reflects the balance between the volatility and stability of EO compounds and the physiological sensitivity of microorganisms. Overall, active packaging proved to be a promising strategy to extend the microbial shelf life of fresh tomato and kale.

### 3.2. Physicochemical Quality

Cherry tomatoes had an initial SSC of 7.5 °Brix (Table 2 (A)), which was similar to those published in the literature for this tomato type [19]. The SSC levels of tomatoes stored under the lower temperatures (10 and 15 °C) remained unchanged (*p* > 0.05) during the 14–15 days of storage. In particular, the SSC of samples were 7.2–8.0 after 14/15 days at 15/10 °C, without differences (*p* > 0.05) among the packaging treatments. No significant SSC changes were observed in tomatoes for the first 5 days at 22 °C, although significantly lower SSC values were observed after 8 days of storage. In particular, tomatoes stored under active packaging had an SSC value that was 1 °Brix lower on day 8 (22 °C) compared to the control samples at the same sampling time. Sugars are consumed as an energy source during the post-harvest life of fruit and vegetables because of product respiration [35]. Such lower sugar contents in samples under the active packaging may be a metabolic response due to possible EO-induced stress. Accordingly, EOs released from active packaging have triggered antioxidant mechanisms (evidenced by higher activities of antioxidant enzymes) in other plant products [16], which may be responsible for the observed sugar consumption as an energy source.

Tomato TA data showed initial values of 0.7% (Table 2 (A)), which were similar to previous studies on cherry tomatoes [19]. The tomato TA values remained unchanged (*p* > 0.05) during the studied storage periods at all three temperatures (10, 15, and 22 °C), without remarkable differences among active and control treatments. Nevertheless, as expected at high temperature, TA decreases of 0.3 and 0.2 TA units were observed for control and active packaging after 8 days at 22 °C. Such mild TA differences among control and active packaging on day 8 (22 °C) may be explained using organic acids as substrates for an increased EO-induced higher respiration [36]. Finally, the pH of tomato samples ranged between 4.1 and 4.5 (similar to those obtained by [19]), without being affected by either storage time or temperature of packaging treatment (Table 2 (A)).

Kale had an initial SSC of 11.1 °Brix (Table 2 (B)), which agrees with the previous literature for this leafy vegetable [37]. The SSC levels remained unchanged after the studied storage periods at different temperatures, making it impossible to observe differences between control and active packaging. Similarly, no glucose, fructose, or sucrose changes were observed in Chinese kale after 10 days at 1 °C [38]. The sugar profile of curly green kale follows this increasing order: d-fructose > d-glucose > sucrose [39]. Increased sugar contents are expected under cold environments as a cryoprotective mechanism, although such enhanced accumulation is rapidly lost after a few days of deacclimation, as observed in other brassicas [40], leading to sugar reduction during the rest of the post-harvest storage period, as previously observed [39]. In addition, no TA and pH differences (*p* > 0.05) were observed during the storage of kale at different temperatures, regardless of the packaging treatment (Table 2 (B)).

**Figure 3:**
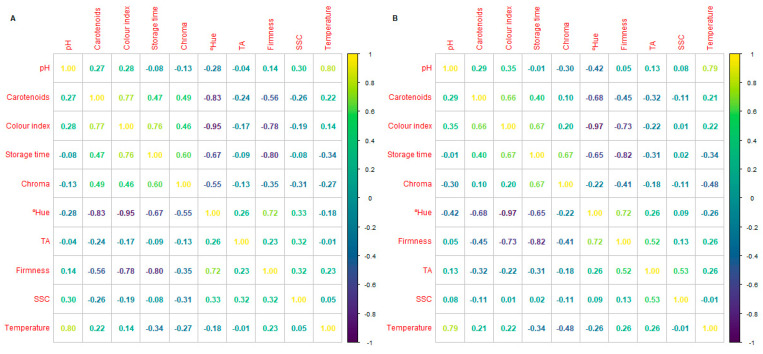
Correlations between firmness, colour indices, carotenes, soluble solid contents (SSCs), pH, titratable acidity (TA), and pH under different storage times and storage temperatures of fresh cherry tomatoes under conventional (**A**) or active packaging (**B**).

**Figure 4:**
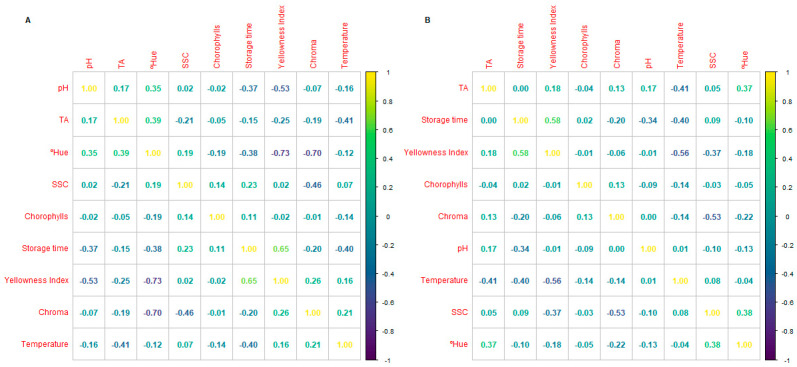
Correlations between firmness, colour indices, carotenes, soluble solid contents (SSCs), pH, titratable acidity (TA), and pH under different storage times and storage temperatures of fresh kale under conventional (**A**) or active packaging (**B**).

In general, no remarkable correlations were observed between the SSC, TA, and pH of kale and tomatoes under different storage times, temperatures, and packaging treatments (Figure 3 and Figure 4). This may be explained by several reasons, which include the following: (i) the used SSC and TA methods are global determinations, which do not allow for finding true correlations of individual sugars and organic acids that occur during the post-harvest life of plant products; (ii) SSC determined by refractometry may also be influenced by the presence of other compounds besides sugars, including acids, amino acids, alcohols, and others; (iii) pH determines the activity of free protons (H^+^), which is not always directly related to the total acid content (titratable acidity), as it depends on the strength and type of the acids present; and (iv) metabolic patterns (respiration, acid-to-sugar conversion, water loss, and plant cell membrane integrity, etc.) may not occur in a linear or simultaneous pattern during the post-harvest life of plant products, which makes it difficult to identify the expected correlations.

### 3.3. Colour and Pigments

The colour indices of tomatoes on day 0 were L*, a*, and b* of 60.9, 51.8, and 82.1, respectively, and the chroma, hue, tomato colour index, a/b and (a/b)^2^ of 97.3, 57.9, 17.6, 0.6, and 0.4, respectively (Table 3 (A), Supplementary Material Table S1). The colour change in tomatoes during post-harvest storage is characterised by a reddening (a* increment), while luminosity (L*) reduction was observed in our samples (Supplementary Materials Table S1), as previously reported [41]. As previously stated, colour indices that integrate those CIELab parameters are more appropriate to describe the colour changes instead of those parameters separately. In particular, refs. [41,42] studied several colour indices and their relationship to the visual colour classification of vine-ripened tomatoes. Among them, the colour index (2000 × a*/L* × (a*^2^ + b*^2^)^0.5^) showed the highest correlation (R^2^ = 0.78; Figure 3) with the rest of the CIELAB parameters to represent the colour changes during tomato storage, as previously reported [42]. A high correlation (0.72) of colour index with the storage time was observed (Figure 3). In particular, the colour index of tomato increased by ≈12 units after 5–6 days at 22 °C and ≈10.5 units after 14 days at 15 °C, regardless (*p* > 0.05) of the packaging treatment at both temperatures. The highest correlation among the quality parameters of tomatoes was observed for colour index (0.72) and firmness (0.80) with storage time, while a high colour index × firmness correlation (−0.78) was also observed (Figure 3).

**Figure 5:**
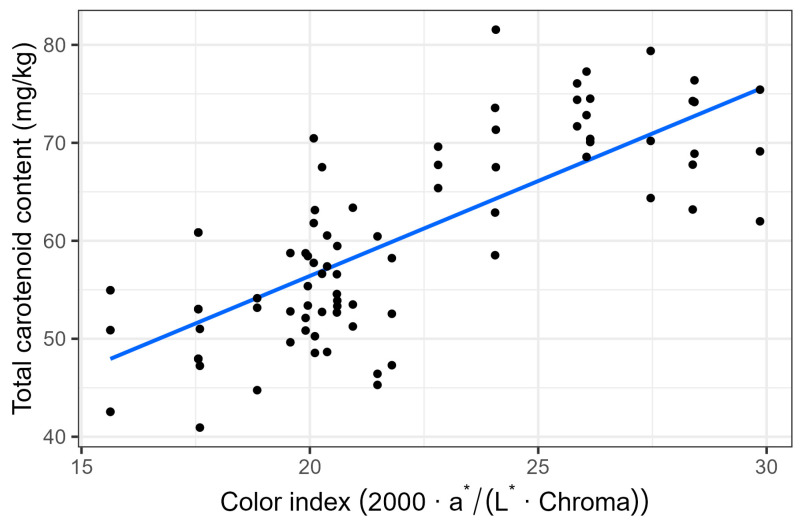
Correlation between total carotene content (mg kg^−1^) and colour index in cherry tomatoes.

The characteristic red colour of ripe tomatoes comes from the synthesis of carotenoids such as lycopene and β-carotene, which are associated during the ripening process with the colour change from green to red in the fruit as the chloroplasts are transformed into chromoplasts [43]. Hence, the initial total carotenoid content (53.9 mg kg^−1^) increased during storage more intensely at higher temperatures with increments of ≈23 units at 22 °C and ≈18 units at 15 °C at the end of the storage periods at those temperatures, regardless (*p* > 0.05) of the packaging treatment (Table 3 (A)). Such a carotenoid increment due to the higher ripening rate at higher temperatures was highly correlated (0.87) with the colour index (Figure 5). Hence, the use of colour index as a non-destructive measurement of cherry tomato quality related to firmness and carotenoid content may be used.

Kale colour data at day 0 were L*, a*, and b* of 48.3, −16.5, and 21.0, respectively, and chroma, hue and YI, of 26.7, 128.6, and 62.7, respectively (Table 3 (B), Supplementary Material Table S2). Colour changes in kale during post-harvest storage are characterised by a greening loss/yellowing, which is linked to the reduction in b* (yellowness) and L* (luminosity) (Supplementary Material Table S2) [13,43]. In particular, L* progressively decreased during storage up to 15–20, while b* increased up to 3–11 units (Table 3 (B)). Hence, YI, which is calculated based on L* and b*, is more appropriate to describe post-harvest colour changes in kale [42,43]. After 7–8 days of storage, YI increased by 2–8 units, without high differences (*p* > 0.05) among storage temperatures and packaging treatments. Nevertheless, and as expected, such increments increased up to 18–22 YI units’ differences at longer storage times (21 days). For packaging treatments, YI increments were reduced by 50% in active samples (comparing YI values of the control and active samples on day 21). Eugenol washing treatment (0.05%) has shown an inhibitory effect on yellowing (lower L* and b* reductions) in fresh-cut lettuce during storage at 4 °C [44]. The last authors demonstrated that this EO component behaved as a competitive inhibitor of the active sites of several colour-degrading enzymes. Similar findings were found by [45], who treated (washing treatment with 0.2–1.5%) fresh-cut Chinese water chestnut with eugenol.

The chlorophyll a and b levels of 1898.4 and 485.9 mg kg^−1^, respectively, were observed in samples on day 0 (Table 3 (B)). As expected, chlorophyll contents decreased during storage, with the highest chlorophyll a reduction of 40% after 7 days at 22 °C (compared to values at day 0) in control samples. However, EOs from active packaging minimised such chlorophyll degradations to reductions of only 7% after 7 days at 22 °C. Reducing storage temperature also lessened the chlorophyll degradation, with a chlorophyll a reduction of 27% and 15% for the control and active samples, respectively, after 21 days at 2 °C. Lower chlorophyll b degradation (<10%) was observed at the end of storage periods at different temperatures, regardless (*p* > 0.05) of packaging treatments. Nevertheless, the observed colour changes were not correlated with chlorophyll contents, which can be explained by several hypotheses: (i) colour in kale is not only attributed to chlorophylls, since kale is very rich in other pigments like the carotenoid lutein, which may be determined more accurately in brassica species by HPLC analyses using C30 columns [46]; (ii) enzymatic degradation products (e.g., pheophytins) of chlorophylls may also absorb in similar spectra to chlorophylls, which may alter the chlorophyll quantifications by spectrophotometric analyses [47]; and (iii) colour measurements are measured on the product surface, while the chlorophyll content is measured in the whole tissue.

Overall, active packaging effectively preserved the tomato and kale colour quality, including pigments (also considered as bioactive compounds) and firmness during storage. In tomatoes, it maintained colour and firmness while supporting natural carotenoid development, showing strong correlations between colour index, firmness, and carotenoids. In kale, it significantly reduced yellowing and chlorophyll degradation—up to 50% less colour loss—thanks to the protective effect of EOs; while in tomatoes, it prevented excessive reddening and over-ripening while supporting the natural increase in carotenoids. Hence, active packaging slowed quality deterioration and extended freshness in both products.

### 3.4. Firmness

Firmness, together with the colour index, was the measured tomato parameter that was highly correlated (0.80 and 0.72, respectively) with the storage time factor (Figure 3). Cherry tomatoes showed an initial firmness of 16.3 N. As expected, at lower storage temperatures (10 and 15 °C), no firmness changes were observed (*p* > 0.05) after 14–15 days, while a firmness reduction of ≈12 N was observed after 8 days at 22 °C. Hence, among the rest of the quality parameters, the highest correlation was observed for firmness × colour index with a value of 0.78 (Figure 3). Hence, firmness together with colour may be considered excellent non-destructive measurements of the product’s quality.

The active packaging did not affect (*p* > 0.05) the firmness of samples at any of the temperatures during storage. However, the firmness of samples under active packaging showed the highest mean values on days 2 and 5 at 22 °C. The plant cell structure, cell wall composition, and intracellular materials of fruit and vegetables are deteriorated during post-harvest life, leading to a reduction in firmness [19,46]. In particular, enzymes (i.e., pectin methylesterase and polygalacturonase) are responsible for such plant cell structure degradation. Active packaging containing carvacrol, oregano EO, and cinnamon EO was shown to inhibit the enzymatic activity of polygalacturonase and polyphenol oxidase in flat peaches [48]. In addition, EOs (thyme EO and rosemary EO) released from active packaging inhibited the key enzymes (aminocyclopropane-1-carboxylic (ACC) oxidase and ACC synthase) of the ethylene biosynthesis pathway [15], which is responsible for plant product maturation, and consequently product softening.

### 3.5. Bioactive Compounds and Total Antioxidant Capacity

#### 3.5.1. Tomatoes: Bioactive Compounds and Total Antioxidant Capacity

The bioactive composition of cherry tomato was evaluated through its total vitamin C, total phenolic content (Folin–Ciocalteu assay), and total antioxidant capacity (DPPH). The antioxidant potential of tomato fruit is usually attributed to its high content of carotenoids—mainly lycopene and β-carotene—together with vitamin C and polyphenols [49,50]. These compounds act synergistically in redox balance and oxidative stress protection during post-harvest storage.

The initial total vitamin C content of tomatoes was 291.9 mg kg^−1^ FW (Table 4 (A)), consistent with the literature values for similar cultivars [51]. During storage, slight fluctuations were observed depending on temperature and packaging treatment. At 10 °C, the vitamin C levels of both control and active packaging remained practically unchanged for 15 days (*p* > 0.05), indicating limited oxidative loss at this low temperature. At 15 °C, values increased moderately by about 5–8% in the first week, followed by a stabilisation phase, while at 22 °C, a slight increase was also observed in active packaging, reaching ≈339 mg kg^−1^ FW after 15 days compared to ≈274 mg kg^−1^ FW in the control samples. This indicates a greater retention of vitamin C under active packaging, particularly at high temperature, probably due to the antioxidant vapour-phase protection provided by the EO compounds, which may limit ascorbate oxidation through reactive oxygen species scavenging [52].

Regarding total phenolic content, determined by the Folin–Ciocalteu method, initial levels were 324.3 mg kg^−1^ FW. Over time, minor variations were recorded, with no statistically significant differences (*p* > 0.05) between packaging treatments at any temperature. At 10 °C, a slight reduction (≈ 10%) in phenolic content was observed after 15 days in both treatments, while at 15 and 22 °C, values remained nearly constant, suggesting that phenolic stability was not compromised under the tested conditions. The retention of these compounds may be related to the low metabolic activity of the fruit at moderate temperatures and the low permeability of the packaging films, which maintain a balanced internal atmosphere, reducing the enzymatic oxidation of phenolics by polyphenol oxidase (PPO).

Antioxidant activity (DPPH) followed a pattern consistent with the evolution of these compounds, but with certain differences depending on temperature. At 10 and 15 °C, the DPPH values showed a rapid initial decrease within the first few days, after which they remained stable until the end of storage. In contrast, at 22 °C, antioxidant capacity remained practically constant, indicating that the higher metabolic rate at this temperature may be balanced by increased synthesis or activation of secondary metabolites with antioxidant potential, such as carotenoids. Importantly, no clear correlation was observed between DPPH and either vitamin C or total phenolic content (R^2^ < 0.50). This suggests that the DPPH response in tomatoes is influenced primarily by lipophilic antioxidants, especially carotenoids, which react differently with the DPPH radical compared with hydrophilic antioxidants. Additionally, the lack of correlation may be due to the kinetics of the DPPH assay, which can be influenced by solvent polarity, extract matrix, and reaction equilibrium time, as previously reported for tomato matrices.

In summary, the results show that active packaging slightly improved vitamin C preservation and maintained stable phenolic levels in cherry tomatoes across all storage temperatures. However, the antioxidant capacity measured by DPPH did not show direct dependence on these hydrophilic antioxidants, emphasising the complexity of redox systems in tomato fruit, where carotenoids and other pigments play a dominant role in antioxidant behaviour.

#### 3.5.2. Kale: Bioactive Compounds and Total Antioxidant Capacity

In kale, the total antioxidant capacity and bioactive compounds were determined through the same analytical parameters: vitamin C, total phenolics, and DPPH. In this leafy vegetable, the antioxidant potential is mainly associated with chlorophylls, carotenoids (especially lutein), vitamin C, and phenolic acids such as caffeic, ferulic, and sinapic acids. The interaction of these compounds defines kale’s resistance to oxidative degradation during post-harvest storage.

The initial vitamin C content was 193.6 mg kg^−1^ FW (Table 4 (B)). During storage, a consistent increase was observed in both treatments, particularly at moderate and high temperatures. In active packaging, vitamin C rose steadily to 265.6 mg kg^−1^ FW after 15 days and remained at 243.2 mg kg^−1^ FW after 21 days, whereas control samples reached only 230.6 mg kg^−1^ FW and 207.8 mg kg^−1^ FW, respectively, at the same times. The retention effect of the active system may be linked to the antioxidant and antimicrobial vapours released from EOs, which limit ascorbate degradation by reducing oxidative enzymatic activity (ascorbate oxidase and peroxidase) and minimising microbial respiration. These findings agree with previous studies reporting that EO-enriched films can delay ascorbate oxidation by maintaining a lower internal O_2_/CO_2_ ratio [53].

The total phenolic content of kale was initially 1471.6 mg kg^−1^ FW, reflecting its naturally high concentration of flavonoids and hydroxycinnamic acids. A decrease was observed during the first storage days after 3 days at 8 °C. However, after longer storage periods (up to 11–15 days), partial recovery or stabilization was detected, with the active packaging showing up to 10–15% higher phenolic values than the control. This suggests a potential inductive or protective effect of EOs, possibly associated with stress responses in plant tissue that trigger phenolic biosynthesis via the phenylpropanoid pathway. Similar behaviours have been observed in fresh-cut lettuce and broccoli treated with thymol or eugenol vapours.

The DPPH antioxidant capacity remained remarkably stable throughout storage, independently of temperature or packaging type. The observed stability suggests that the reactive antioxidant pool in kale did not undergo significant oxidative depletion, likely because of the high inherent antioxidant buffering capacity of its pigment and polyphenol matrix. However, this stability also resulted in no apparent correlation between DPPH and either vitamin C, total phenolics, or chlorophyll content. Indeed, the correlation coefficients between DPPH and these parameters were close to zero, indicating that the DPPH variability observed was within the experimental noise range. The constancy of DPPH may also be due to the assay’s limited sensitivity to subtle shifts in complex matrices rich in chlorophylls and lipophilic pigments, which may interfere with the radical’s absorbance signal. Consequently, even though vitamin C and phenolics showed dynamic changes, their influence on total DPPH reactivity could not be accurately detected.

Overall, the results confirm that active packaging effectively reduced oxidative degradation in kale, particularly preserving vitamin C and phenolic contents during extended storage. Despite the absence of a direct quantitative relationship with DPPH, these components are known to contribute individually to antioxidant defence mechanisms. The weak DPPH responsiveness, therefore, reflects methodological limitations rather than an absence of antioxidant function.

## 4. Conclusions

The effectiveness of using EOs on microbial growth was observed, as it was reduced in both products during storage. This effect was greater at higher temperatures for bacteria and at lower temperatures for moulds. Regarding physicochemical quality, the active packaging preserved firmness and colour in cherry tomatoes. In kale, yellowing and, therefore, chlorophyll degradation were reduced, preserving the product’s freshness. The active packaging contributed to bioactive compounds such as vitamin C, which were enhanced by greater retention at high temperatures thanks to the antioxidant action of the EOs in the vapour phase. Phenolic compounds showed a small increase in kale due to a stress response induced by the EOs. Therefore, active packaging with EOs increases product shelf life by preserving quality during storage. Further, combining the preservation of fruit and vegetable products in active packaging to preserve microbiological and physicochemical quality and increase shelf life, together with predictive models to avoid non-destructive analysis, can be a major breakthrough in post-harvest preservation. Future research includes exploring alternative natural antimicrobials, incorporating smart functionalities and advancing sustainable materials to broaden industrial applicability.

## Figures and Tables

**Table 1:** (A) Parameter estimates (±standard error of regression) for the Baranyi–Ratkowsky model describing the growth of different microbial groups on fresh cherry tomatoes under control or active packaging during storage at 10, 15, and 22 °C. (B). Parameter estimates (±standard error of regression) for the Baranyi–Ratkowsky model describing the growth of different microbial groups on fresh kale under control or active packaging during storage at 10, 15, and 22 °C.

**(A)**						
**Microorganism**	**Packaging**	$T_{min}$ **(°C)**	***b*** **(°C^−1^)**	$\log N_{0}$ **(log CFU/g)**	$\log N_{max}$ **(log CFU/g)**	$\log C_{0}$ **(·)**
Mesophiles	Active	11.3 ± 0.69	0.16 ± 0.03	2.22 ± 0.09	4.84 ± 0.24	^†^
	Control	6.65 ± 1.16	0.10 ± 0.01	2.39 ± 0.15	5.65 ± 0.29	^†^
Enterobacteria	Active	10.27 ± 0.73	0.11 ± 0.01	2.22 ± 0.06	4.50 ± 0.16	^†^
	Control	10.75 ± 0.51	0.14 ± 0.01	2.40 ± 0.08	6.49 ± 0.20	^†^
Yeasts	Active	−4.36 ± 4.66	0.04 ± 0.01	2.32 ± 0.14	5.14 ± 0.96	0.49 ± 2.6
	Control	−1.09 ± 3.36	0.05 ± 0.01	2.38 ± 0.15	5.53 ± 0.52	^†^
Moulds	Active	−3.66 ± 1.57	0.04 ± 0.01	1.95 ± 0.03	3.71 ± 0.10	1.34 ± 0.4
	Control	−9.41 ± 6.58	0.03 ± 0.01	2.00 ± 0.11	4.59 ± 0.81	^†^
Psychrophiles	Active	19.76 ± 0.01	0.58 ± 0.01	2.60 ± 0.12	^‡^	^†^
	Control	8.51 ± 0.96	0.11 ± 0.01	2.58 ± 0.13	7.85 ± 0.46	^†^
**(B)**						
**Microorganism**	**Packaging**	$T_{min}$ **(°C)**	***b*** **(°C^−1^)**	$\log N_{0}$ **(log CFU/g)**	$\log N_{max}$ **(log CFU/g)**	$\log C_{0}$ **(·)**
Mesophiles	Active	−3.47 ± 0.88	0.11 ± 0.01	3.43 ± 0.18	7.48 ± 0.31	^†^
	Control	−4.99 ± 0.93	0.10 ± 0.01	3.48 ± 0.17	7.75 ± 0.18	^†^
Enterobacteria	Active	−4.59 ± 0.38	0.18 ± 0.01	2.78 ± 0.14	8.29 ± 0.23	^†^
	Control	−3.47 ± 0.62	0.13 ± 0.01	2.25 ± 0.17	7.53 ± 0.19	^†^
Yeasts	Active	−7.81 ± 1.09	0.07 ± 0.01	2.66 ± 0.12	6.79 ± 0.16	^†^
	Control	−6.10 ± 0.78	0.10 ± 0.01	2.62 ± 0.11	6.64 ± 0.12	^†^
Moulds	Active	−1.84 ± 0.86	0.06 ± 0.01	2.53 ± 0.10	7.33 ± 0.38	−0.35 ± 0.51
	Control	−4.30 ± 1.04	0.06 ± 0.01	2.57 ± 0.11	7.52 ± 0.41	^†^
Psychrophiles	Active	−2.92 ± 1.03	0.07 ± 0.01	3.92 ± 0.13	8.58 ± 0.26	^†^
	Control	2.59 ± 0.67	0.12 ± 0.01	4.32 ± 0.15	9.97 ± 0.30	^†^

^†^ The experiment did not have a lag phase at any temperature based on the AIC test. ^‡^ The experiment did not reach a stationary phase at any temperature.

**Table 2:** (A) Soluble solid content, pH, and titratable acidity of fresh cherry tomato packaged under control or active packaging during storage at 10, 15, and 22 °C (n = 3 ± SD). Capital letters denote significant (*p* < 0.05) differences among packaging treatments for the same sampling time. Lowercase letters denote significant (*p* < 0.05) differences among sampling times for the same packaging treatment. (B). Soluble solid content, pH, and titratable acidity of fresh kale packaged under control or active packaging during storage at 2, 8, 15, and 22 °C (n = 3 ± SD). Capital letters denote significant (*p* < 0.05) differences among packaging treatments for the same sampling time. Lowercase letters denote significant (*p* < 0.05) differences among sampling times for the same packaging treatment.

**(A)**						
	**SSC**		**pH**		**TA**	
	Control	Active	Control	Active	Control	Active
**Initial**	7.5 ± 0.2 ^Aa^	7.5 ± 0.2 ^Aa^	4.1 ± 0.0 ^Aa^	4.1 ± 0.0 ^Aa^	0.7 ± 0.1 ^Aa^	0.7 ± 0.1 ^Aa^
**10 °C**						
4	8.3 ± 0.5 ^Aa^	7.9 ± 1.0 ^Aa^	4.2 ± 0.0 ^Aa^	4.2 ± 0.0 ^Aa^	0.7 ± 0.2 ^Aa^	0.9 ± 0.1 ^Aa^
8	8.3 ± 1.5 ^Aa^	8.1 ± 0.2 ^Aa^	4.2 ± 0.1 ^Aa^	4.2 ± 0.1 ^Aa^	0.6 ± 0.0 ^Aa^	0.7 ± 0.1 ^Aa^
10	8.0 ± 1.0 ^Aa^	7.4 ± 0.4 ^Aa^	4.2 ± 0.1 ^Aa^	4.2 ± 0.0 ^Aa^	0.7 ± 0.0 ^Aa^	0.7 ± 0.1 ^Aa^
15	7.9 ± 1.1 ^Aa^	7.5 ± 0.7 ^Aa^	4.2 ± 0.0 ^Aa^	4.1 ± 0.0 ^Aa^	0.7 ± 0.0 ^Aa^	0.8 ± 0.2 ^Aa^
**15 °C**						
3	8.0 ± 1.2 ^Aa^	7.9 ± 1.3 ^Aa^	4.3 ± 0.1 ^Aa^	4.3 ± 0.0 ^Aa^	0.7 ± 0.0 ^Aa^	0.8 ± 0.1 ^Aa^
7	7.7 ± 0.1 ^Aa^	7.4 ± 0.8 ^Aa^	4.3 ± 0.1 ^Aa^	4.3 ± 0.1 ^Aa^	0.6 ± 0.0 ^Aa^	0.7 ± 0.1 ^Aa^
9	8.0 ± 0.7 ^Aa^	8.4 ± 0.6 ^Aa^	4.3 ± 0.1 ^Aa^	4.3 ± 0.1 ^Aa^	0.7 ± 0.0 ^Aa^	0.7 ± 0.1 ^Aa^
14	7.2 ± 0.3 ^Aa^	7.7 ± 0.3 ^Aa^	4.4 ± 0.0 ^Aa^	4.5 ± 0.1 ^Aa^	0.5 ± 0.0 ^Aa^	0.5 ± 0.1 ^Aa^
**22 °C**						
1	7.3 ± 0.3 ^Ba^	8.2 ± 0.6 ^Aa^	4.3 ± 0.0 ^Aa^	4.3 ± 0.0 ^Aa^	0.7 ± 0.0 ^Aa^	0.7 ± 0.0 ^Aa^
2	8.7 ± 0.2 ^Aa^	6.4 ± 0.3 ^Ba^	4.4 ± 0.0 ^Aa^	4.2 ± 0.0 ^Aa^	0.7 ± 0.0 ^Aa^	0.7 ± 0.0 ^Aa^
5	8.5 ± 0.2 ^Aa^	7.8 ± 0.5 ^Ba^	4.3 ± 0.0 ^Aa^	4.3 ± 0.1 ^Aa^	0.7 ± 0.0 ^Aa^	0.7 ± 0.0 ^Aa^
8	7.1 ± 0.3 ^Aa^	5.1 ± 0.5 ^Ba^	4.3 ± 0.1 ^Aa^	4.2 ± 0.1 ^Aa^	0.2 ± 0.0 ^Aa^	0.5 ± 0.0 ^Aa^
**(B)**						
	**SSC**		**pH**		**TA**	
	Control	Active	Control	Active	Control	Active
**Initial**	11.1 ± 0.1 ^Ab^	11.1 ± 0.1 ^Aab^	6.3 ± 0.1 ^Aa^	6.3 ± 0.1 ^Aa^	0.5 ± 0.0 ^Aa^	0.7 ± 0.0 ^Aa^
**2 °C**						
3	11.1± 0.2 ^Aa^	11.0 ± 0.1 ^Aab^	6.2 ± 0.2 ^Aa^	6.3 ± 0.1 ^Aa^	0.5 ± 0.0 ^Aa^	0.5 ± 0.0 ^Aa^
8	11.0 ± 0.1 ^Ab^	10.9 ± 0.1 ^Ab^	6.2 ± 0.1 ^Aa^	6.3 ± 0.1 ^Aa^	0.5 ± 0.0 ^Aa^	0.5 ± 0.0 ^Aa^
11	11.3 ± 0.2 ^Aa^	11.1 ± 0.1 ^Aa^	6.3 ± 0.1 ^Aa^	6.3 ± 0.0 ^Aa^	0.5 ± 0.0 ^Aa^	0.5 ± 0.0 ^Aa^
15	11.1 ± 0.2 ^Ab^	11.1 ± 0.1 ^Aa^	6.2 ± 0.1 ^Aa^	6.2 ± 0.1 ^Aa^	0.5 ± 0.0 ^Aa^	0.5 ± 0.0 ^Aa^
21	10.9 ± 0.2 ^Ab^	11.0 ± 0.1 ^Aab^	6.3 ± 0.1 ^Aa^	6.2 ± 0.1 ^Aa^	0.5 ± 0.0 ^Aa^	0.5 ± 0.0 ^Aa^
**8 °C**						
2	11.2 ± 0.1 ^Aa^	11.1 ± 0.1 ^Aa^	6.3 ± 0.1 ^Aa^	6.3 ± 0.1 ^Aa^	0.5 ± 0.0 ^Aa^	0.6 ± 0.0 ^Aa^
7	11.1 ± 0.1 ^Aa^	11.3 ± 0.2 ^Aa^	6.3 ± 0.1 ^Aa^	6.2 ± 0.1 ^Aa^	0.5 ± 0.0 ^Aa^	0.5 ± 0.0 ^Aa^
10	11.0 ± 0.2 ^Aa^	11.2 ± 0.2 ^Aa^	6.3 ± 0.1 ^Aa^	6.3 ± 0.1 ^Aa^	0.5 ± 0.0 ^Aa^	0.5 ± 0.0 ^Aa^
14	11.1 ± 0.1 ^Aa^	11.2 ± 0.1 ^Aa^	6.3 ± 0.0 ^Aa^	6.3 ± 0.0 ^Aa^	0.5 ± 0.0 ^Aa^	0.5 ± 0.0 ^Aa^
16	11.1± 0.2 ^Aa^	11.1± 0.1 ^Aa^	6.2 ± 0.1 ^Aa^	6.3 ± 0.0 ^Aa^	0.5 ± 0.0 ^Aa^	0.5 ± 0.0 ^Aa^
**15 °C**						
2	11.7± 0.3 ^Aa^	10.9 ± 0.1 ^Aa^	5.9 ± 0.2 ^Aa^	6.1 ± 0.1 ^Aa^	0.5 ± 0.0 ^Aa^	0.4 ± 0.0 ^Aa^
4	11.7 ± 0.1 ^Aa^	10.9 ± 0.1 ^Aa^	6.4 ± 0.2 ^Aa^	5.8 ± 0.2 ^Aa^	0.5 ± 0.0 ^Aa^	0.4 ± 0.0 ^Aa^
7	11.8 ± 0.2 ^Aa^	11.4± 0.3 ^Aa^	6.3 ± 0.2 ^Aa^	6.0 ± 0.1 ^Aa^	0.5 ± 0.1 ^Aa^	0.4 ± 0.1 ^Aa^
9	11.7 ± 0.3 ^Aa^	11.6 ± 0.2 ^Aa^	6.0 ± 0.1 ^Aa^	6.2 ± 0.1 ^Aa^	0.5 ± 0.0 ^Aa^	0.4 ± 0.0 ^Aa^
**22 °C**						
1	11.2 ± 0.2 ^Aa^	11.3 ± 0.2 ^Aa^	6.2 ± 0.1 ^Aa^	6.3 ± 0.2 ^Aa^	0.5 ± 0.0 ^Aa^	0.5 ± 0.0 ^Aa^
2	11.4 ± 0.1 ^Aa^	11.3 ± 0.2 ^Aa^	6.3 ± 0.2 ^Aa^	6.3 ± 0.1 ^Aa^	0.5 ± 0.0 ^Aa^	0.6 ± 0.0 ^Aa^
3	10.9 ± 0.1 ^Aa^	11.2 ± 0.2 ^Aa^	6.2 ± 0.1 ^Aa^	6.3 ± 0.2 ^Aa^	0.5 ± 0.0 ^Aa^	0.5 ± 0.0 ^Aa^
4	11.1 ± 0.1 ^Aa^	10.9 ± 0.2 ^Aa^	6.2 ± 0.2 ^Aa^	6.2 ± 0.2 ^Aa^	0.5 ± 0.0 ^Aa^	0.5 ± 0.0 ^Aa^
7	11.1± 0.1 ^Aa^	11.0± 0.1 ^Aa^	6.2 ± 0.1 ^Aa^	6.2 ± 0.1 ^Aa^	0.5 ± 0.0 ^Aa^	0.5 ± 0.0 ^Aa^

**Table 3:** (A) Colour and pigments (total carotenoid content) of fresh cherry tomatoes packaged under control or active packaging during storage at 10, 15, and 22 °C (n = 3 ± SD). Capital letters denote significant (*p* < 0.05) differences among packaging treatments for the same sampling time. Lowercase letters denote significant (*p* < 0.05) differences among sampling times for the same packaging treatment. (B) Colour and pigments (total chlorophylls) of fresh kale packaged under control or active packaging during storage at 2, 8, 15, and 22 °C (n = 3 ± SD). Capital letters denote significant (*p* < 0.05) differences among packaging treatments for the same sampling time. Lowercase letters denote significant (*p* < 0.05) differences among sampling times for the same packaging treatment.

**(A)**													
	**Initial**	**10 °C**				**15 °C**					**22 °C**		
		**4**	**8**	**10**	**15**	**3**	**7**	**9**	**14**	**1**	**2**	**5**	**8**
**Chroma** Control	97.3 ± 6.6 ^Aa^	96.1 ± 8.7 ^Aa^	103.9 ± 7.2 ^Aa^	106.2 ± 7.0 ^Aa^	105.4 ± 4.9 ^Aa^	97.7 ± 7.8 ^Aa^	96.8 ± 7.1 ^Aa^	103.1 ± 4.2 ^Aa^	99.2 ± 3.5 ^Aa^	89.3 ± 8.9 ^Aa^	108.7 ± 8.4 ^Aa^	83.7 ± 7.0 ^Aa^	111.0 ± 6.1 ^Aa^
Active	97.3 ± 6.6 ^Aa^	99.3 ± 9.8 ^Aa^	102.2 ± 7.2 ^Aa^	101.8 ± 7.2 ^Aa^	101.2 ± 6.1 ^Aa^	97.4 ± 8.5 ^Aa^	105.3 ± 6.9 ^Aa^	104.0 ± 7.3 ^Aa^	102.8 ± 6.7 ^Aa^	96.0 ± 9.9 ^Aa^	114.1 ± 7.0 ^Aa^	83.2 ± 5.1 ^Aa^	107.4 ± 6.1 ^Aa^
**Hue** Control	57.9 ± 3.5 ^Aa^	54.2 ± 3.9 ^Aa^	54.6 ± 3.3 ^Aa^	54.0 ± 3.8 ^Aa^	52.4 ± 2.9 ^Aa^	51.9 ± 4.1 ^Aa^	48.8 ± 2.3 ^Aa^	48.4 ± 3.3 ^Aa^	44.3 ± 2.0 ^Aa^	52.3 ± 6.9 ^Aa^	39.8 ± 4.2 ^Aa^	37.2 ± 2.1 ^Aa^	35.0 ± 1.8 ^Aa^
Active	57.9 ± 3.5 ^Aa^	54.9 ± 2.9 ^Aa^	54.4 ± 3.8 ^Aa^	52.6 ± 3.3 ^Aa^	54.8 ± 4.2 ^Aa^	54.8 ± 4.5 ^Aa^	51.2 ± 2.9 ^Aa^	49.9 ± 3.6 ^Aa^	46.2 ± 2.5 ^Aa^	56.7 ± 6.9 ^Aa^	42.0 ± 4.2 ^Aa^	39.2 ± 3.7 ^Aa^	34.4 ± 2.1 ^Aa^
**Colour index** Control	17.6 ± 2.7 ^Aa^	21.8 ± 2.8 ^Aa^	24.1 ± 1.4 ^Aa^	25.9 ± 3.4 ^Aa^	28.4 ± 1.2 ^Aa^	21.8 ± 2.8 ^Aa^	24.1 ± 1.4 ^Aa^	25.9 ± 3.4 ^Aa^	28.4 ± 1.2 ^Aa^	22.7 ± 5.1 ^Aa^	28.1 ± 3.0 ^Aa^	57.0 ± 2.0 ^Aa^	43.4 ± 1.6 ^Aa^
Active	17.6 ± 2.7 ^Aa^	19.9 ± 2.7 ^Aa^	22.8 ± 2.3 ^Aa^	24.1 ± 3.2 ^Aa^	27.5 ± 2.3 ^Aa^	19.9 ± 2.7 ^Aa^	22.8 ± 2.3 ^Aa^	24.1 ± 3.2 ^Aa^	27.5 ± 2.3 ^Aa^	19.8 ± 4.5 ^Aa^	25.8 ± 2.5 ^Aa^	51.5 ± 3.3 ^Aa^	43.7 ± 1.4 ^Aa^
**a/b** Control	0.6 ± 0.1 ^Aa^	0.7 ± 0.1 ^Aa^	0.7 ± 0.1 ^Aa^	0.7 ± 0.1 ^Aa^	0.8 ± 0.1 ^Aa^	0.8 ± 0.1 ^Aa^	0.9 ± 0.1 ^Aa^	0.9 ± 0.1 ^Aa^	1.0 ± 0.1 ^Aa^	0.8 ± 0.2 ^Aa^	1.3 ± 0.1 ^Aa^	1.5 ± 0.1 ^Aa^	1.6 ± 0.1 ^Aa^
Active	0.6 ± 0.1 ^Aa^	0.7 ± 0.1 ^Aa^	0.7 ± 0.1 ^Aa^	0.8 ± 0.1 ^Aa^	0.7 ± 0.1 ^Aa^	0.7 ± 0.1 ^Aa^	0.8 ± 0.1 ^Aa^	0.8 ± 0.1 ^Aa^	1.0 ± 0.1 ^Aa^	0.7 ± 0.2 ^Aa^	1.2 ± 0.1 ^Aa^	1.4 ± 0.1 ^Aa^	1.7 ± 0.1 ^Aa^
**(a/b)^2^** Control	0.4 ± 0.1 ^Aa^	0.5 ± 0.2 ^Aa^	0.5 ± 0.1 ^Aa^	0.5 ± 0.2 ^Aa^	0.6 ± 0.1 ^Aa^	0.6 ± 0.2 ^Aa^	0.8 ± 0.1 ^Aa^	0.8 ± 0.2 ^Aa^	1.1 ± 0.1 ^Aa^	0.7 ± 0.3 ^Aa^	1.7 ± 0.2 ^Aa^	2.0 ± 0.1 ^Aa^	2.4 ± 0.1 ^Aa^
Active	0.4 ± 0.1 ^Aa^	0.5 ± 0.2 ^Aa^	0.5 ± 0.2 ^Aa^	0.6 ± 0.2 ^Aa^	0.5 ± 0.2 ^Aa^	0.5 ± 0.2 ^Aa^	0.7 ± 0.1 ^Aa^	0.7 ± 0.2 ^Aa^	0.9 ± 0.2 ^Aa^	0.5 ± 0.2 ^Aa^	1.4 ± 0.2 ^Aa^	1.8 ± 0.2 ^Aa^	2.6 ± 0.1 ^Aa^
**Total carotenoids (mg kg^−1^)** Control	53.9 ± 6.5 ^Aa^	54.0 ± 8.0 ^Aa^	5.9 ± 7.7 ^Aa^	54.6 ± 2.0 ^Aa^	50.7 ± 8.4 ^Aa^	52.7 ± 5.5 ^Aa^	65.0 ± 7.7 ^Aa^	74.0 ± 2.2 ^Aa^	73.1 ± 3.8 ^Aa^	50.7 ± 5.2 ^Aa^	56.0 ± 6.4 ^Aa^	68.8 ± 6.7 ^Aa^	72.9 ± 4.4 ^Aa^
Active	53.9 ± 6.5 ^Aa^	53.7 ± 4.6 ^Aa^	55.7 ± 2.5 ^Aa^	55.6 ± 3.4 ^Aa^	55.5 ± 6.2 ^Aa^	53.9 ± 4.2 ^Aa^	67.6 ± 2.1 ^Aa^	73.5 ± 7.3 ^Aa^	71.3 ± 7.6 ^Aa^	46.4 ± 5.1 ^Aa^	63.3 ± 6.5 ^Aa^	68.4 ± 5.6 ^Aa^	71.7 ± 2.5 ^Aa^
**(B)**													
	**Initial**	**2 °C**					**8 °C**						
		**3**	**8**	**11**	**15**	**21**	**2**		**7**	**10**	**14**	**16**	
**Chroma** Control	26.7 ± 6.0 ^Aa^	19.8 ± 4.1 ^Aa^	27.5 ± 8.5 ^Aa^	16.8 ± 6.6 ^Aa^	27.9 ± 8.3 ^Aa^	24.7 ± 8.4 ^Aa^	20.5 ± 4.4 ^Aa^		17.5 ± 4.7 ^Aa^	22.5 ± 8.9 ^Aa^	16.1 ± 3.5 ^Aa^	22.1 ± 6.4 ^Aa^	
Active	26.7 ± 6.0 ^Aa^	19.8 ± 7.9 ^Aa^	18.0 ± 4.8 ^Aa^	19.9 ± 6.6 ^Aa^	23.5 ± 8.7 ^Aa^	28.4 ± 9.8 ^Aa^	21.4 ± 5.1 ^Aa^		17.5 ± 4.0 ^Aa^	16.0 ± 4.7 ^Aa^	26.9 ± 6.1 ^Aa^	22.5 ± 7.8 ^Aa^	
**Hue** Control	128.6 ± 2.3 ^Aa^	128.5 ± 2.7 ^Aa^	129.4 ± 10.7 ^Aa^	130.3 ± 3.5 ^Aa^	129.7 ± 11.9 ^Aa^	124.0 ± 6.2 ^Aa^	135.2 ± 28.1 ^Aa^		130.2 ± 2.2 ^Aa^	126.0 ± 3.8 ^Ab^	129.2 ± 3.4 ^Ab^	127.2 ± 5.4 ^Ab^	
Active	128.6 ± 2.3 ^Aa^	132.0 ± 6.9 ^Aa^	131.5 ± 4.3 ^Aa^	128.4 ± 4.8 ^Aa^	126.1 ± 3.1 ^Aa^	121.1 ± 8.7 ^Aa^	136.5 ± 25.2 ^Aa^		129.8 ± 3.4 ^Aa^	130.0 ± 3.8 ^Aa^	124.3 ± 3.2 ^Aa^	127.2 ± 5.5 ^Aa^	
**Total colour difference** Control	9.8 ± 5.2 ^Aa^	22.4 ± 6.7 ^Aa^	19.8 ± 5.6 ^Aa^	23.1 ± 9.2 ^Aa^	16.7 ± 8.2 ^Aa^	18.5 ± 7.0 ^Aa^	16.6 ± 7.3 ^Aa^		23.6 ± 7.0 ^Aa^	20.8 ± 8.9 ^Aa^	21.9 ± 8.9 ^Aa^	12.8 ± 7.1 ^Aa^	
Active	9.8 ± 5.2 ^Aa^	19.7 ± 6.4 ^Aa^	20.6± 6.8 ^Aa^	17.4 ± 10.4 ^Aa^	15.8 ± 7.2 ^Aa^	16.5 ± 8.1 ^Aa^	14.8 ± 9.8 ^Aa^		21.7 ± 7.9 ^Aa^	22.6 ± 9.9 ^Aa^	13.4 ± 7.3 ^Aa^	17.7 ± 9.4 ^Aa^	
**Yellowness Index** Control	62.7 ± 13.0 ^Aa^	82.9 ± 18.7 ^Aa^	92.7 ± 24.9 ^Aa^	65.8 ± 20.0 ^Aa^	85.6 ± 23.7 ^Aa^	86.0 ± 21.4 ^Aa^	65.7 ± 12.2 ^Ab^		71.4 ± 12.2 ^Ab^	85.6 ± 15.6 ^Aa^	62.5 ± 13.8 ^Ac^	66.0 ± 23.5 ^Ab^	
Active	62.7± 13.0 ^Aa^	62.6 7 ± 23.4 ^Aa^	65.3 ± 18.8 ^Aa^	66.8 ± 9.8 ^Aa^	74.3 ± 16.3 ^Aa^	74.8 ± 19.6 ^Aa^	63.4 ± 12.1 ^Ab^		68.0 ± 14.8 ^Ab^	63.4 ± 16.3 ^Ab^	85.1 ± 15.5 ^Aa^	79.5 ± 26.6 ^Ab^	
**Total chlorophylls (mg kg^−1^)** Control	2026.1 ± 71.1 ^Aa^	2065.0 ± 323.4 ^Aa^	2079.0 ± 120.4 ^Aa^	1867.9± 122.9 ^Aa^	1845.9 ± 113.1 ^Aa^	2068.0 ± 144.3 ^Aa^	192.6 ± 203.2 ^Aa^		2024.7 ± 135.7 ^Aa^	2194.2 ± 138.4 ^Aa^	1863.7 ± 74.2 ^Aa^	1999.6 ± 155.8 ^Aa^	
Active	2026.1 ± 71.1 ^Aa^	2144.5 ± 107.9 ^Aa^	1883.2 ± 138.2 ^Aa^	2385.1 ± 241.7 ^Aa^	1941.3 ± 92.2 ^Aa^	2040.0 ± 90.3 ^Aa^	2040.0 ± 42.5 ^Aa^		1832.1 ± 81.1 ^Aa^	1984.5 ± 243.2 ^Aa^	2072.6 ± 83.9 ^Aa^	2017.9 ± 97.3 ^Aa^	
	**15 °C**				**22 °C**								
Days	**2**	**4**	**7**	**9**	**1**	**2**	**3**		**4**	**7**			
**Chroma** Control	21.5 ± 4.5 ^Aa^	11.5 ± 4.7 ^Aa^	12.3 ± 5.7 ^Aa^	18.9 ± 4.5 ^Aa^	20.2 ± 5.5 ^Aa^	17.0 ± 5.0 ^Aa^	12.3 ± 5.7 ^Aa^		18.9 ± 4.5 ^Aa^	18.3 ± 6.4 ^Aa^			
Active	28.9 ± 6.5 ^Aa^	131.8 ± 4.6 ^Aa^	11.5 ± 4.0 ^Aa^	31.6 ± 8.2 ^Aa^	16.5 ± 4.2 ^Aa^	23.6 ± 7.3 ^Aa^	11.5 ± 4.7 ^Aa^		32.7 ± 4.6 ^Aa^	18.9 ± 7.8 ^Aa^			
**Hue** Control	129.9 ± 3.2 ^Aba^	131.8 ± 2.2 ^Aa^	128.9 ± 2.4 ^Ab^	129.9 ± 2.9 ^Ab^	133.3 ± 6.1 ^Aa^	128.3 ± 7.5 ^Aa^	128.9 ± 2.4 ^Aa^		129.9 ± 2.9 ^Aa^	125.9 ± 5.4 ^Aa^			
Active	128.2 ± 3.6 ^Ab^	118.4 ± 4.6 ^Ac^	131.1 ± 3.4 ^Aa^	126.8 ± 4.5 ^Ab^	133.4 ± 2.7 ^Aa^	127.5 ± 3.4 ^Aa^	131.8 ± 2.2 ^Aa^		118.4 3.3 ^Aa^	125.8 ± 5.5 ^Aa^			
**Total colour difference** Control	9.5 ± 5.3 ^Ba^	57.2 ± 7.0 ^Aa^	42.1 ± 9.6 ^Ba^	18.3 ± 7.4 ^Aa^	16.4 ± 7.1 ^Aa^	20.5 ± 7.0 ^Aa^	42.1 ± 9.9 ^Aa^		18.3 ± 7.4 ^Aa^	16.8 ± 7.1 ^Aa^			
Active	12.3 ± 4.8 ^Aa^	14.5 ± 5.8 ^Ba^	48.4 ± 7.9 ^Aa^	10.2 ± 5.5 ^Ba^	16.3 ± 5.7 ^Aa^	14.2 ± 5.6 ^Aa^	57.2 ± 7.0 ^Aa^		14.5 ± 5.8 ^Aa^	32.2 ± 9.4 ^Aa^			
**Yellow** Control	52.5 ± 14.8 ^Aa^	81.4 ± 12.2 ^Aa^	73.6 ± 15.3 ^Aa^	50.5 ± 10.5 ^Aa^	40.7 ± 15.6 ^Aa^	60.1 ± 10.4 ^Aa^	73.6 ± 15.3 ^Aa^		50.5 ± 10.5 ^Aa^	69.5 ± 23.5 ^Aa^			
Active	69.9 ± 15.1 ^Aa^	132.7 ± 19.3 ^Aa^	73.7 ± 14.8 ^Aa^	67.8 ± 16.4 ^Aa^	47.7 ± 12.1 ^Aa^	70.8 ± 17.6 ^Aa^	81.4 ± 12.2 ^Aa^		132.7 ± 19.3 ^Aa^	100.8 ± 26.6 ^Aa^			
**Total chlorophylls** Control	2082.7 ± 151.7 ^Aa^	1985.8 ± 252.2 ^Aa^	1792.7 ± 49.8 ^Aa^	1980.2 ± 180.3 ^Aa^	2152.1 ± 128.9 ^Aa^	2001.7 ± 259.1 ^Aa^	2041.7± 152.4 ^Aa^		1930.5 ± 76.9 ^Aa^	2135.4 ± 31.8 ^Aa^			
Active	1661.4 344.2 ^Aa^	2080.9 ± 457.2 ^Aa^	1850.6 ± 39.3 ^Aa^	1882.8 ± 27.9 ^Aa^	2122.8 ± 169.7 ^Aa^	2257.8 ± 169.8 ^Aa^	1844.0 ± 24.1 ^Aa^		1945.4 ± 43.1 ^Aa^	2403.7 ± 79.9 ^Aa^			

**Table 4:** (A) Total contents of vitamin C, phenolic compounds, carotenes, and total antioxidant capacity of fresh cherry tomatoes packaged under control or active packaging during storage at 10, 15, and 22 °C (n = 3 ± SD). Capital letters denote significant (*p* < 0.05) differences among packaging treatments for the same sampling time. Lowercase letters denote significant (*p* < 0.05) differences among sampling times for the same packaging treatment. (B) Total contents of vitamin C, phenolic compounds, chlorophylls, and total antioxidant capacity of fresh kale packaged under control or active packaging during storage at 2, 8, 15, and 22 °C (n = 3 ± SD). Capital letters denote significant (*p* < 0.05) differences among packaging treatments for the same sampling time. Lowercase letters denote significant (*p* < 0.05) differences among sampling times for the same packaging treatment.

**(A)**						
	**Total Phenolic Content** **(mg kg^−1^ FW)**		**Antioxidant Activity** **(mmol kg^−1^ FW)**		**Vitamin C** **(mg kg^−1^ FW)**	
	Control	Active	Control	Active	Control	Active
**Initial**	324.3 ± 24.3 ^Aa^	324.3 ± 24.3 ^Aa^	2.6 ± 0.4 ^Aa^	2.6 ± 0.4 ^Aa^	291.9 ± 77.7 ^Aa^	291.9 ± 77.7 ^Aa^
**10 °C**						
4	332.2 ± 49.8 ^Aa^	321.7 ± 14.4 ^Aa^	3.7 ± 0.5 ^Aa^	3.7 ± 0.2 ^Aa^	252.5 ± 62.2 ^Aa^	251.3 ± 30.6 ^Aa^
8	314.5 ± 2.5 ^Aa^	304.6 ± 9.6 ^Aa^	3.5 ± 0.3 ^Aa^	3.7 ± 0.1 ^Aa^	254.2 ± 56.8 ^Aa^	284.1 ± 37.6 ^Aa^
10	327.2 ± 2.9 ^Aa^	346.6 ± 47.2 ^Aa^	3.7 ± 0.3 ^Aa^	3.5 ± 0.4 ^Aa^	310.0 ± 70.8 ^Aa^	313.5 ± 55.1 ^Aa^
15	350.7 ± 31.8 ^Aa^	295.2 ± 12.0 ^Aa^	3.5 ± 0.4 ^Aa^	4.2 ± 0.4 ^Aa^	273.9 ± 24.4 ^Aa^	339.1 ± 17.5 ^Aa^
**15 °C**						
3	371.0 ± 29.3 ^Aa^	417.1 ± 62.2 ^Aa^	2.5 ± 0.1 ^Aa^	2.5 ± 0.3 ^Aa^	194.7 ± 17.2 ^Aa^	290.4 ± 10.3 ^Aa^
7	383.4 ± 36.4 ^Aa^	366.1 ± 33.6 ^Aa^	2.9 ± 0.3 ^Aa^	2.6 ± 0.1 ^Aa^	273.1 ± 41.1 ^Aa^	243.7 ± 40.8 ^Aa^
9	389.3 ± 26.5 ^Aa^	403.8 ± 60.1 ^Aa^	2.6 ± 0.1 ^Aa^	2.6 ± 0.2 ^Aa^	298.7 ± 47.1 ^Aa^	310.5 ± 18.4 ^Aa^
14	419.1 ± 62.3 ^Aa^	403.2 ± 50.4 ^Aa^	2.7 ± 0.1 ^Aa^	2.6 ± 0.2 ^Aa^	264.8 ± 19.6 ^Aa^	286.4 ± 44.8 ^Aa^
**22 °C**						
1	326.1 ± 34.2 ^Aa^	324.6 ± 81.7 ^Aa^	2.2 ± 0.1 ^Aa^	2.1 ± 0.2 ^Aa^	165.2 ± 58.6 ^Aa^	151.1 ± 11.1 ^Aa^
2	310.8 ± 17.2 ^Aa^	334.7 ± 25.8 ^Aa^	2.3 ± 0.2 ^Aa^	2.2 ± 0.1 ^Aa^	313.7 ± 35.7 ^Aa^	236.7 ± 62.7 ^Aa^
5	396.0 ± 36.4 ^Aa^	324.2 ± 28.8 ^Aa^	2.4 ± 0.2 ^Aa^	2.4 ± 0.1 ^Aa^	182.9 ± 30.3 ^Aa^	215.8 ± 17.0 ^Aa^
8	382.2 ± 12.4 ^Aa^	379.4 ± 63.5 ^Aa^	2.4 ± 0.1 ^Aa^	2.4 ± 0.2 ^Aa^	191.0 ± 27.8 ^Aa^	228.6 ± 6.0 ^Aa^
**(B)**						
	**Total Phenolic Content** **(mg kg^−1^ FW)**		**Antioxidant Activity** **(mmol kg^−1^ FW)**		**Vitamin C** **(mg kg^−1^ FW)**	
	Control	Active	Control	Active	Control	Active
**Initial**	1471.6 ± 19.4 ^Aa^	1471.6 ± 224.2 ^Aa^	3.3 ± 0.2 ^Aa^	3.3 ± 0.2 ^Aa^	193.6 ± 7.1 ^Aa^	193.6 ± 7.1 ^Aa^
**2 °C**						
3	1252.5 ± 224.2 ^Aa^	1304.9 ± 95.5 ^Aa^	3.1 ± 0.4 ^Aa^	3.6 ± 0.2 ^Aa^	183.9 ± 18.8 ^Aa^	254.5 ± 10.0 ^Aa^
8	1023.8 ± 114.6 ^Aa^	1032.3 ± 33.8 ^Aa^	2.9 ± 0.2 ^Aa^	2.8 ± 0.2 ^Aa^	264.6 ± 16.1 ^Aa^	294.8 ± 11.0 ^Aa^
11	902.5 ± 115.8 ^Aa^	1750.5 ± 165.5 ^Aa^	3.5 ± 0.7 ^Aa^	3.9 ± 0.1 ^Aa^	226.3 ± 16.8 ^Aa^	260.8 ± 14.5 ^Aa^
15	1591.5 ± 36.4 ^Aa^	924.9 ± 215.5 ^Aa^	3.7 ± 0.6 ^Aa^	2.7 ± 0.2 ^Aa^	230.6 ± 14.9 ^Aa^	265.6 ± 35.9 ^Aa^
21	1494.3 ± 221.4 ^Aa^	1190.2 ± 126.0 ^Aa^	3.2 ± 0.2 ^Aa^	3.6 ± 0.2 ^Aa^	207.8 ± 12.8 ^Aa^	243.2 ± 46.0 ^Aa^
**8 °C**						
2	1298.1 ± 206.5 ^Aa^	1423.2 ± 9.1 ^Aa^	2.9 ± 0.1 ^Aa^	2.6 ± 0.1 ^Aa^	182.8 ± 5.5 ^Aa^	263.1 ± 35.1 ^Aa^
7	1548.0 ± 101.0 ^Aa^	1098.8 ± 76.3 Aa	2.9 ± 0.3 ^Aa^	2.9 ± 0.1 ^Aa^	233.9 ± 27.8 ^Aa^	226.7 ± 18.2 ^Aa^
10	1098.4 ± 30.1 ^Aa^	993.3 ± 35.4 ^Aa^	3.5 ± 0.1 ^Aa^	3.7 ± 0.1 ^Aa^	274.3 ± 46.4 ^Aa^	254.2 ± 24.7 ^Aa^
14	874.1 ± 131.1 ^Aa^	1165.7 ± 104.0 ^Aa^	3.8 ± 0.0 ^Aa^	3.4 ± 0.2 ^Aa^	200.4 ± 21.7 ^Aa^	288.8 ± 25.5 ^Aa^
16	1167.2 ± 141.1 ^Aa^	1263.9 ± 17.2 ^Aa^	2.7 ± 0.1 ^Aa^	3.1 ± 0.2 ^Aa^	205.4 ± 22.9 ^Aa^	243.3 ± 12.8 ^Aa^
**15 °C**						
2	1133.7 ± 34.3 ^Aa^	1200.6 ± 104.2 ^Aa^	3.1 ± 0.6 ^Aa^	3.7 ± 0.1 ^Aa^	410.3 ± 51.8 ^Aa^	127.1 ± 12.8 ^Aa^
4	1280.3 ± 209.6 ^Aa^	961.5 ± 172.9 ^Aa^	3.4 ± 0.1 ^Aa^	2.7 ± 0.2 ^Aa^	185.2 ± 60.3 ^Aa^	221.1 ± 27.7 ^Aa^
7	1394.6 ± 131.2 ^Aa^	1554.8 ± 67.0 ^Aa^	2.7 ± 0.2 ^Aa^	3.0 ± 0.0 ^Aa^	177.8 ± 9.0 ^Aa^	171.7 ± 19.2 ^Aa^
9	926.0 ± 210.3 ^Aa^	1580.4 ± 94.8 ^Aa^	2.7 ± 0.1 ^Aa^	3.7 ± 0.2 ^Aa^	320.4 ± 31.7 ^Aa^	412.4 ± 44.3 ^Aa^
**22 °C**						
1	1514.3 ± 111.5 ^Aa^	1524.3 ± 251.3 ^Aa^	3.8 ± 0.3 ^Aa^	3.8 ± 0.7 ^Aa^	297.4 ± 32.9 Aa	250.4 ± 31.8 ^Aa^
2	1662.5 ± 63.5 ^Aa^	1656.3 ± 54.7 ^Aa^	3.7 ± 0.2 ^Aa^	4.0 ± 0.1 ^Aa^	244.2 ± 24.1 Aa	379.4 ± 21.5 ^Aa^
3	1504.9 ± 180.0 ^Aa^	1495.6 ± 69.2 ^Aa^	3.7 ± 0.2 ^Aa^	3.4 ± 0.2 ^Aa^	411.1 ± 59.6 Aa	326.7 ± 40.8 ^Aa^
4	1351.4 ± 132.0 ^Aa^	1575.3 ± 161.0 ^Aa^	3.3 ± 0.2 ^Aa^	3.6 ± 0.2 ^Aa^	339.4 ± 48.5 Aa	371.1 ± 36.2 ^Aa^
7	1806.8 ± 435.9 ^Aa^	1582.3 ± 49.4 ^Aa^	3.6 ± 0.8 ^Aa^	3.1 ± 0.8 ^Aa^	162.4 ± 9.7 Aa	225.0 ± 3.3 ^Aa^

## Data Availability

The original contributions presented in this study are included in the article/Supplementary Materials. Further inquiries can be directed to the corresponding author.

## Supplementary Materials

The following supporting information can be downloaded at https://www.mdpi.com/article/10.3390/foods14244225/s1, Figure S1: Baranyi-Ratkowsky model (-) fitted to the growth on tomatoes of microorganisms (mesophiles, enterobacteria, yeast, molds or psychrophiles) under active or conventional packaging conditions at 10, 15 and 22 °C; Figure S2: Baranyi-Ratkowsky model (-) fitted to the growth on kale of microorganisms (mesophiles, enterobacteria, yeast, molds or psychrophiles) under active or conventional packaging conditions at 10, 15 and 22 °C; Table S1. Colour parameters (L*, a* and b*) of fresh cherry tomatoes packaged under control or active packaging during storage at 10, 15 and 22 °C (n = 3 ± SD). Capital letters denote significant (*p* < 0.05) differences among packaging treatments for the same sampling time. Lowercase letters denote significant (*p* < 0.05) differences among sampling times for the same packaging treatment; Table S2. Colour parameters (L*, a* and b*) of fresh kale packaged under control or active packaging during storage at 2, 80, 15 and 22 °C (n = 3 ± SD). Capital letters denote significant (*p* < 0.05) differences among packaging treatments for the same sampling time. Lowercase letters denote significant (*p* < 0.05) differences among sampling times for the same packaging treatment.
