# Supplementary material for: Effects of Active Paper Sheets on the Quality of Cherry Tomatoes and Kale During Storage
Source: Foods. 2025 Dec 9;14(24):4225. doi: 10.3390/foods14244225 (PMC12733043; doi:10.3390/foods14244225)
Supplement: Supplementary file 1 [file foods-14-04225-s001.zip › CAPTIONS - Supplementary Material.pdf]

Supplementary Material Figure S1: Baranyi-Ratkowsky model (-) fitted to the growth on tomatoes of microorganisms (mesophiles, enterobacteria, yeast, molds or psychrophiles) under active or conventional packaging conditions at 10, 15 and 22 °C.

Supplementary Material Figure S2: Baranyi-Ratkowsky model (-) fitted to the growth on kale of microorganisms (mesophiles, enterobacteria, yeast, molds or psychrophiles) under active or conventional packaging conditions at 10, 15 and 22 °C.

Supplementary Material Table S1. Colour parameters (*L**, *a** and *b**) of fresh cherry tomatoes packaged under control or active packaging during storage at 10, 15 and 22 °C ($n = 3 \pm SD$). Capital letters denote significant ($p < 0.05$) differences among packaging treatments for the same sampling time. Lowercase letters denote significant ($p < 0.05$) differences among sampling times for the same packaging treatment.

Supplementary Material Table S2. Colour parameters (*L**, *a** and *b**) of fresh kale packaged under control or active packaging during storage at 2, 80, 15 and 22 °C ($n = 3 \pm SD$). Capital letters denote significant ($p < 0.05$) differences among packaging treatments for the same sampling time. Lowercase letters denote significant ($p < 0.05$) differences among sampling times for the same packaging treatment.
