# Supplementary material for: Effects of Active Paper Sheets on the Quality of Cherry Tomatoes and Kale During Storage
Source: Foods. 2025 Dec 9;14(24):4225. doi: 10.3390/foods14244225 (PMC12733043; doi:10.3390/foods14244225)
Supplement: Supplementary file 1 [file foods-14-04225-s001.zip › Supplementary Material Table S1.pdf]

Supplementary Material Table S1. Colour parameters (*L**, *a** and *b**) of fresh cherry tomatoes packaged under control or active packaging during storage at 10, 15 and 22 °C (n = 3 ± SD). Capital letters denote significant ($p < 0.05$) differences among packaging treatments for the same sampling time. Lowercase letters denote significant ($p < 0.05$) differences among sampling times for the same packaging treatment.

	Initial	10 °C				15 °C				22 °C			
		4	8	10	15	3	7	9	14	1	2	5	8
L* Control	60.9 ± 3.9 Aa	58.3 ± 2.8 Aa	57.4 ± 3.4 Aa	57.3 ± 3.1 Aa	57.0 ± 2.8 Aa	56.8 ± 2.8 Aa	54.8 ± 2.6 Aa	51.9 ± 5.2 Aa	50.3 ± 1.5 Aa	57.4 ± 6.9 Aa	63.5 ± 3.6 Aa	33.1± 2.2 Aa	45.8 ± 2.5 Aa
Active	60.9± 3.9 Aa	58.8 ± 2.3 Aa	58.5 ± 3.0 Aa	58.6 ± 4.0 Aa	56.8 ± 3.3 Aa	58.0 ± 2.7 Aa	55.2 ± 2.8 Aa	53.9 ± 3.7 Aa	50.5 ± 2.4 Aa	56.7 ± 4.2 Aa	64.7 ± 2.7 Aa	34.9± 3.3 Aa	46.2 ± 2.1 Aa
a* Control	51.8 ± 7.6 Aa	56.1 ± 6.9 Aa	60.1 ± 5.9 Aa	62.3 ± 6.7 Aa	64.2 ± 7.5 Aa	60.4 ± 9.0 Aa	63.7 ± 5.1 Aa	68.4 ± 4.2 Aa	70.9 ± 2.0 Aa	56.1 ± 11.1 Aa	96.6 ± 9.2 Aa	78.9 ± 3.1 Aa	110.7 ± 3.6 Aa
Active	51.8± 7.6 Aa	57.1 ± 7.8 Aa	59.3 ± 7.0 Aa	61.0 ± 6.7 Aa	58.2 ± 6.2 Aa	56.0 ± 7.6 Aa	65.9 ± 5.2 Aa	66.7 ± 5.0 Aa	71.0 ± 4.2 Aa	54.1± 13.2 Aa	95.3± 7.7 Aa	74.9± 6.1 Aa	108.9 ± 4.2 Aa
b* Control	82.1 ± 3.8 Aa	77.8 ± 6.9 Aa	84.6 ± 4.6 Aa	85.7 ± 7.0 Aa	83.4 ± 4.8 Aa	76.6 ± 5.1 Aa	72.8 ± 6.2 Aa	77.0 ± 6.0 Aa	69.4 ± 4.5 Aa	70.6 ± 8.5 Aa	71.6 ± 6.0 Aa	53.0 ± 7.0 Aa	67.1± 5.9 Aa
Active	82.1 ± 3.8 Aa	81.1 ± 8.2 Aa	82.9 ± 5.6 Aa	80.8 ± 6.6 Aa	82.5 ± 6.9 Aa	79.4 ± 8.3 Aa	82.0 ± 7.1 Aa	79.5 ± 8.4 Aa	74.2 ± 7.0 Aa	79.4 ± 6.7 Aa	78.5 ± 6.8 Aa	54.4 ± 4.6 Aa	63.9 ± 5.7 Aa
