# Supplementary material for: Effects of Active Paper Sheets on the Quality of Cherry Tomatoes and Kale During Storage
Source: Foods. 2025 Dec 9;14(24):4225. doi: 10.3390/foods14244225 (PMC12733043; doi:10.3390/foods14244225)
Supplement: Supplementary file 1 [file foods-14-04225-s001.zip › Supplementary Material Table S2.pdf]

Supplementary Material Table S2. Colour parameters (*L**, *a**, and *b**) of fresh kale packaged under control or active packaging during storage at 2, 80, 15, and 22 °C (n = 3 ± SD). Capital letters denote significant ($p < 0.05$) differences among packaging treatments for the same sampling time. Lowercase letters denote significant ($p < 0.05$) differences among sampling times for the same packaging treatment.

	Initial	2 °C					8 °C				
		3	8	11	15	21	2	7	10	14	16
**L*** Control	48.3 ± 9.6 ^{Aa}	27.3 ± 6.1 ^{Ba}	32.7 ± 9.8 ^{Aa}	28.6 ± 9.7 ^{Ba}	35.6 ± 9.8 ^{Aa}	34.8 ± 11.4 ^{Ba}	35.1 ± 6.0 ^{Aa}	26.8 ± 7.0 ^{Aa}	29.7 ± 9.1 ^{Aa}	29.5 ± 8.9 ^{Aa}	39.5 ± 8.5 ^{Aa}
Active	48.3 ± 9.6 ^{Aa}	32.6 ± 7.5 ^{Aa}	30.4 ± 4.6 ^{Aa}	33.2 ± 9.5 ^{Aa}	35.8 ± 8.1 ^{Aa}	46.7 ± 14.7 ^{Aa}	37.3 ± 8.4 ^{Aa}	28.8 ± 6.2 ^{Aa}	28.9 ± 9.8 ^{Aa}	38.0 ± 9.4 ^{Aa}	33.7 ± 10.4 ^{Aa}
**a*** Control	-16.5 ± 2.9 ^{Aa}	-12.3 ± 2.5 ^{Aa}	-16.5 ± 3.5 ^{Aa}	-10.8 ± 3.7 ^{Aa}	-16.7 ± 3.4 ^{Aa}	-13.3 ± 3.3 ^{Aa}	-10.9 ± 7.0 ^{Aa}	-11.2 ± 2.6 ^{Aa}	-12.9 ± 4.5 ^{Aa}	-10.1 ± 2.3 ^{Aa}	-13.0 ± 3.1 ^{Aa}
Active	-16.5 ± 2.9 ^{Aa}	-12.0± 3.7 ^{Aa}	-11.7± 2.6 ^{Aa}	-12.4 ± 4.2 ^{Aa}	-13.7 ± 4.6 ^{Aa}	-13.7 ± 3.5 ^{Aa}	-11.6 ± 8.0 ^{Aa}	-11.2 ± 2.7 ^{Aa}	-10.2± 2.7 ^{Aa}	-14.9 ± 2.7 ^{Aa}	-13.1 ± 3.6 ^{Aa}
**b*** Control	21.0 ± 5.4 ^{Aa}	15.5 ± 3.4 ^{Aa}	21.5 ± 9.2 ^{Aa}	12.9 ± 5.6 ^{Aa}	21.7 ± 9.2 ^{Aa}	20.7 ± 8.2 ^{Aa}	16.1 ± 4.2 ^{Aa}	13.5 ± 4.0 ^{Aa}	18.4 ± 7.9 ^{Aa}	12.5 ± 2.9 ^{Aa}	17.8 ± 6.0 ^{Aa}
Active	21.0 ± 5.4 ^{Aa}	14.3 ± 7.3 ^{Aa}	13.6 ± 4.2 ^{Aa}	15.6 ± 5.4 ^{Aa}	19.0 ± 7.5 ^{Aa}	24.5 ± 10.2 ^{Aa}	16.4 ± 4.5 ^{Aa}	13.4 ± 3.2 ^{Aa}	12.3 ± 4.0 ^{Aa}	22.3 ± 5.7 ^{Aa}	18.1 ± 7.2 ^{Aa}

	15 °C				22 °C				
Days	2	4	7	9	1	2	3	4	7
**L*** Control	47.5 ± 9.8 ^{Aa}	14.8 ± 8.7 ^{Aa}	20.0 ± 11.2 ^{Aa}	43.9 ± 14.1 ^{Aa}	55.3 ± 12.6 ^{Aa}	31.6 ± 10.0 ^{Ba}	20.0 ± 11.2 ^{Aa}	43.9 ± 14.1 ^{Aa}	32.2 ± 8.5 ^{Aa}
Active	47.3 ± 10.2 ^{Aa}	33.2 ± 10.1 Aa	17.2 ± 7.2 ^{Aa}	45.3 ± 7.6 ^{Aa}	37.6 ± 10.6 ^{Ba}	38.6 ± 7.1 ^{Aa}	14.8 ± 5.9 ^{Ba}	33.2 ± 8.5 ^{Ba}	23.5 ± 10.4 ^{Ba}
**a*** Control	-13.7 ± 2.3 ^{Aa}	-7.6 ± 2.6 ^{Aa}	-7.8 ± 3.6 ^{Aa}	-12.1 ± 2.6 ^{Aa}	-13.5 ± 2.8 ^{Aa}	-10.5 ± 3.7 ^{Aa}	-7.8 ± 3.6 ^{Aa}	-12.1 ± 2.6	-18.5 ± 3.1 ^{Aa}
Active	-17.8 ± 3.7 ^{Aa}	-15.7 ± 1.9 ^{Aa}	-7.6 ± 2.7 ^{Aa}	-18.2 ± 3.5 ^{Aa}	-11.3 ± 2.4 ^{Aa}	-13.9 ± 3.4 ^{Aa}	-7.6 ± 2.6 ^{Aa}	-15.7 ± 1.9 ^{Aa}	-37.2 ± 3.6 ^{Aa}
**b*** Control	16.5 ± 4.1 ^{Aa}	8.6 ± 4.0 ^{Aa}	9.5 ± 4.5 ^{Aa}	14.5 ± 3.8 ^{Aa}	14.9 ± 5.3 ^{Aa}	13.1 ± 4.2 ^{Aa}	9.5 ± 4.5 ^{Aa}	14.5 ± 3.8 ^{Aa}	24.1 ± 6.0 ^{Aa}
Active	22.7 ± 5.6 ^{Aa}	29.2 ± 4.5 ^{Aa}	8.6 ± 3.2 ^{Aa}	25.7 ± 7.7 ^{Aa}	12.0 ± 3.5 ^{Aa}	19.0 ± 6.6 ^{Aa}	8.6 ± 4.0 ^{Aa}	29.2 ± 4.5 ^{Aa}	41.6 ± 7.2 ^{Aa}
