# Supplementary figures and images for: Effects of Active Paper Sheets on the Quality of Cherry Tomatoes and Kale During Storage
Source: Foods. 2025 Dec 9;14(24):4225. doi: 10.3390/foods14244225 (PMC12733043; doi:10.3390/foods14244225)

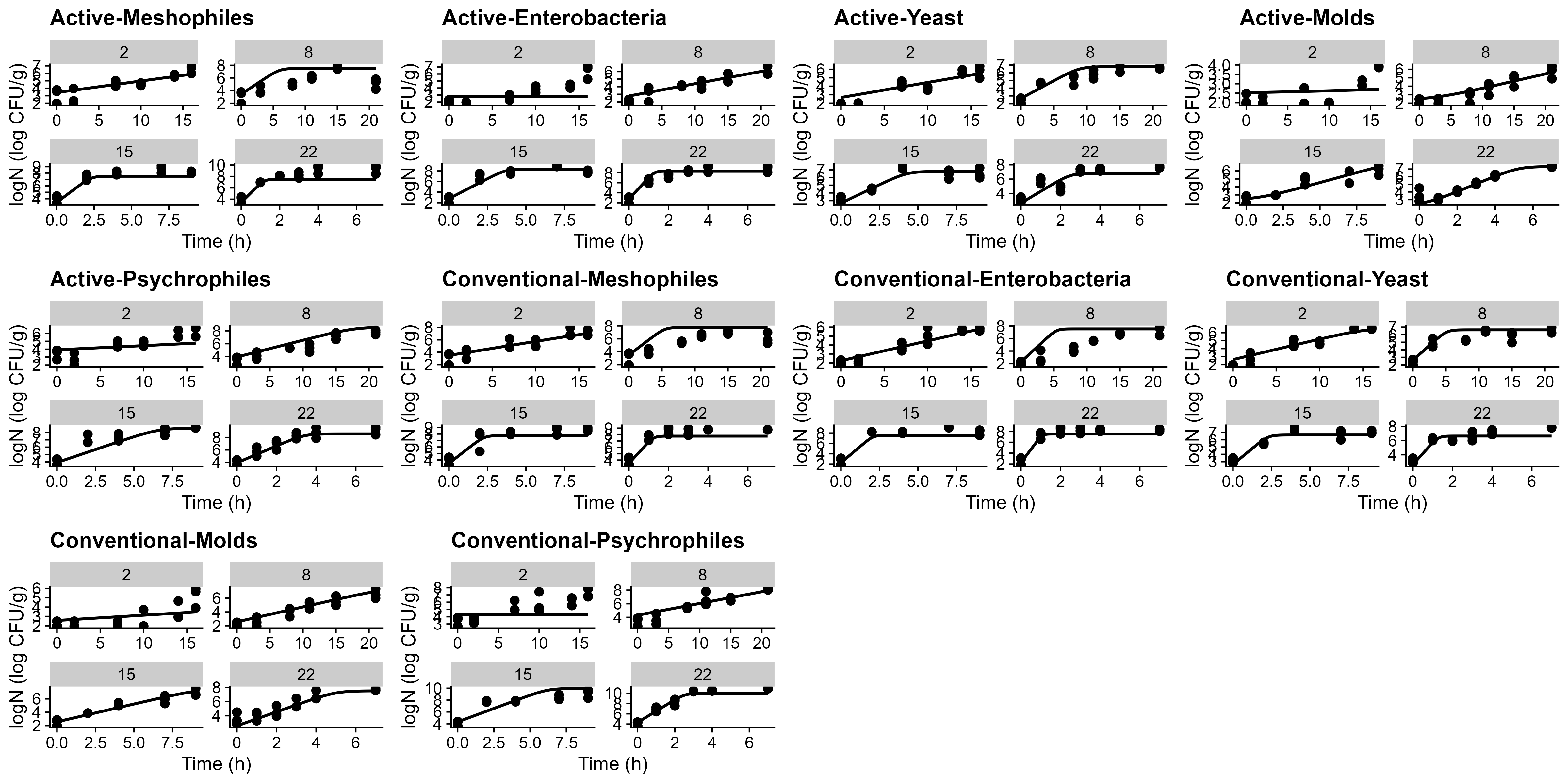

Supplement: Supplementary file 1 [file foods-14-04225-s001.zip › Supplementary Material Figure S2.png]

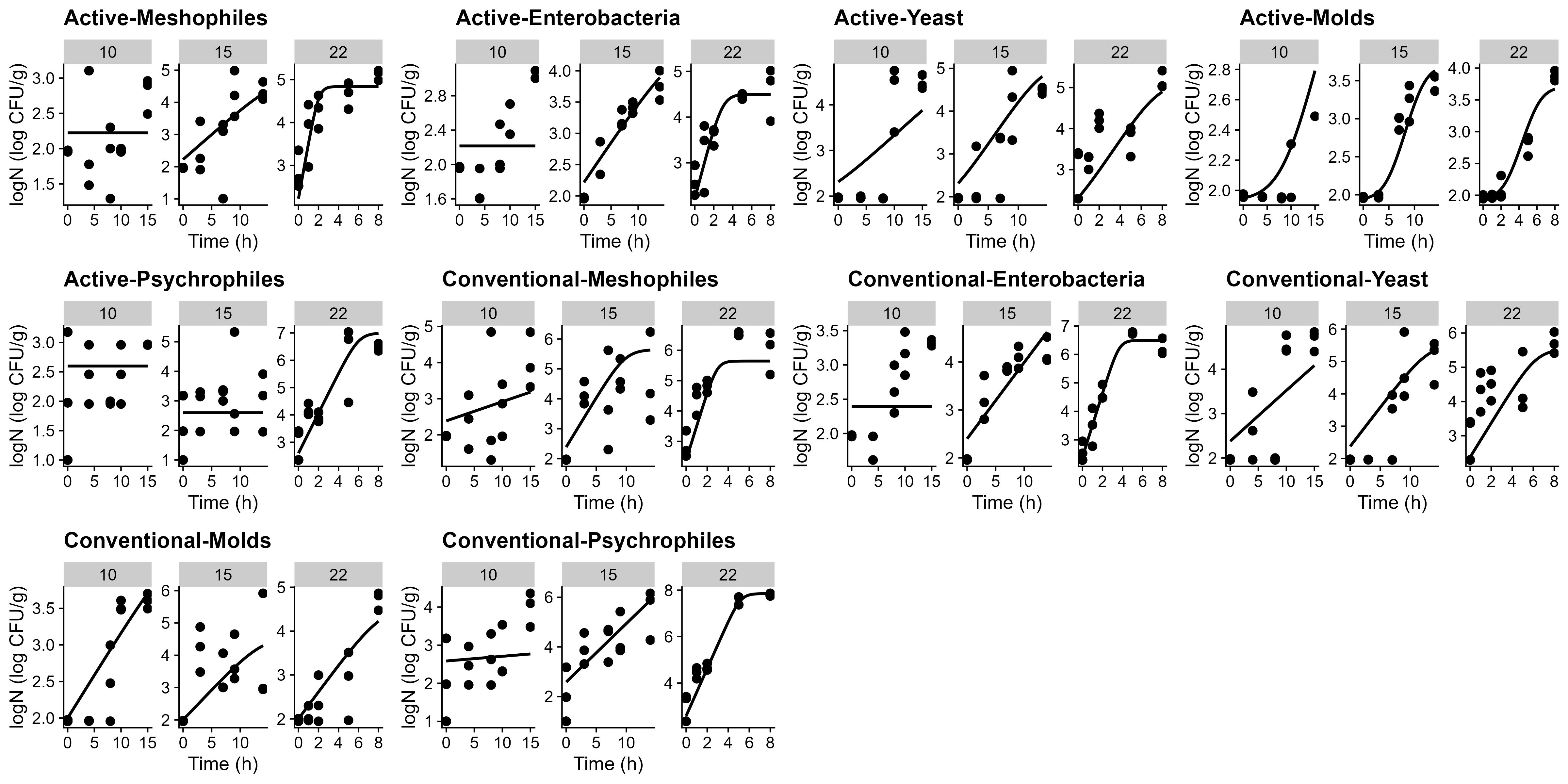

Supplement: Supplementary file 1 [file foods-14-04225-s001.zip › Supplementary Material Figure S1.png]
